# Supplementary figures and images for: Cross-device federated unsupervised learning for the detection of anomalies in single-lead electrocardiogram signals
Source: PLOS Digit Health. 2025 Apr 7;4(4):e0000793. doi: 10.1371/journal.pdig.0000793 (PMC11975069; doi:10.1371/journal.pdig.0000793)

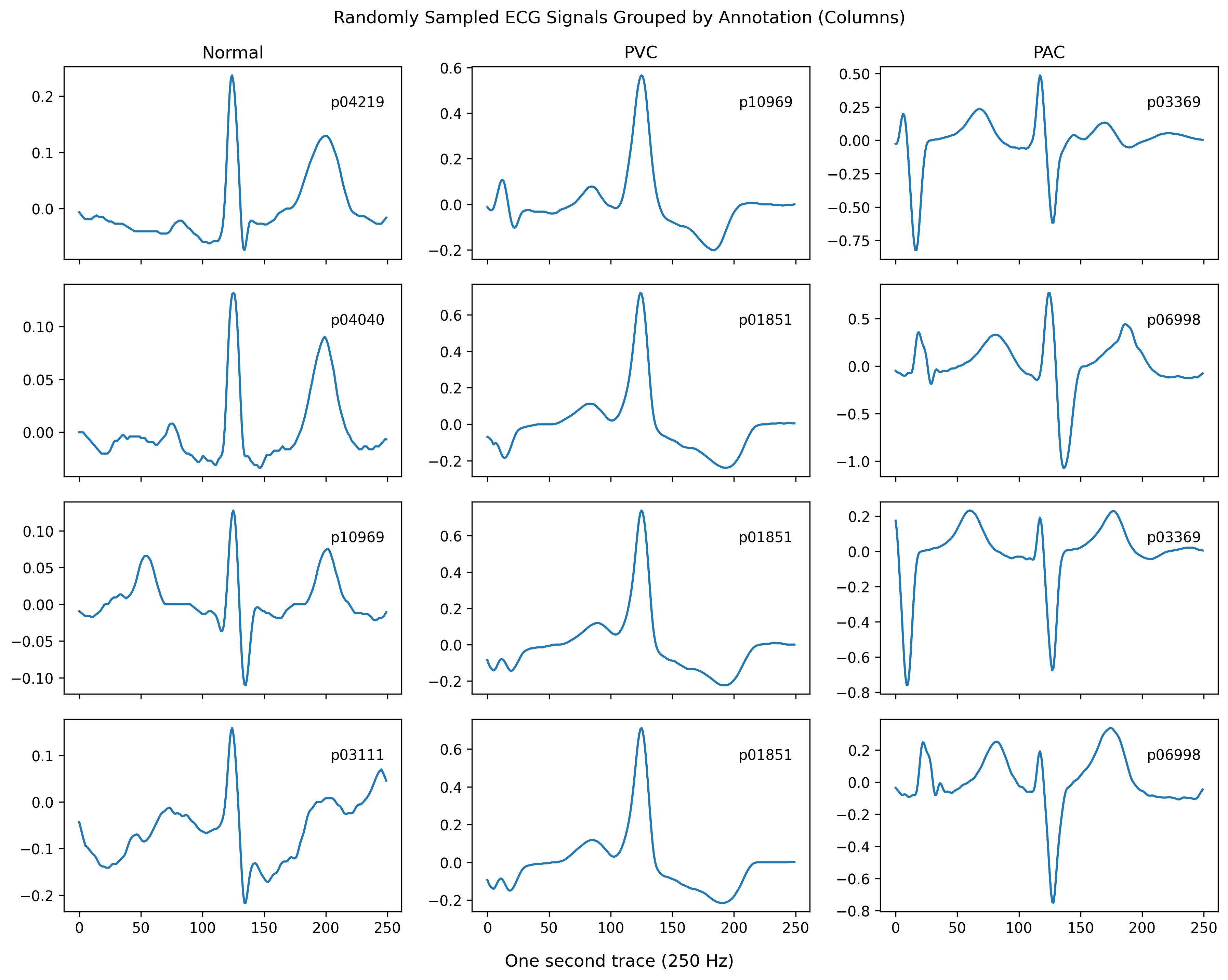

Supplement: S1 Fig — Twelve randomly sampled ECG traces to illustrate the data and demonstrate the impact of pathologies on the waveform. (PNG) [file pdig.0000793.s001.png]

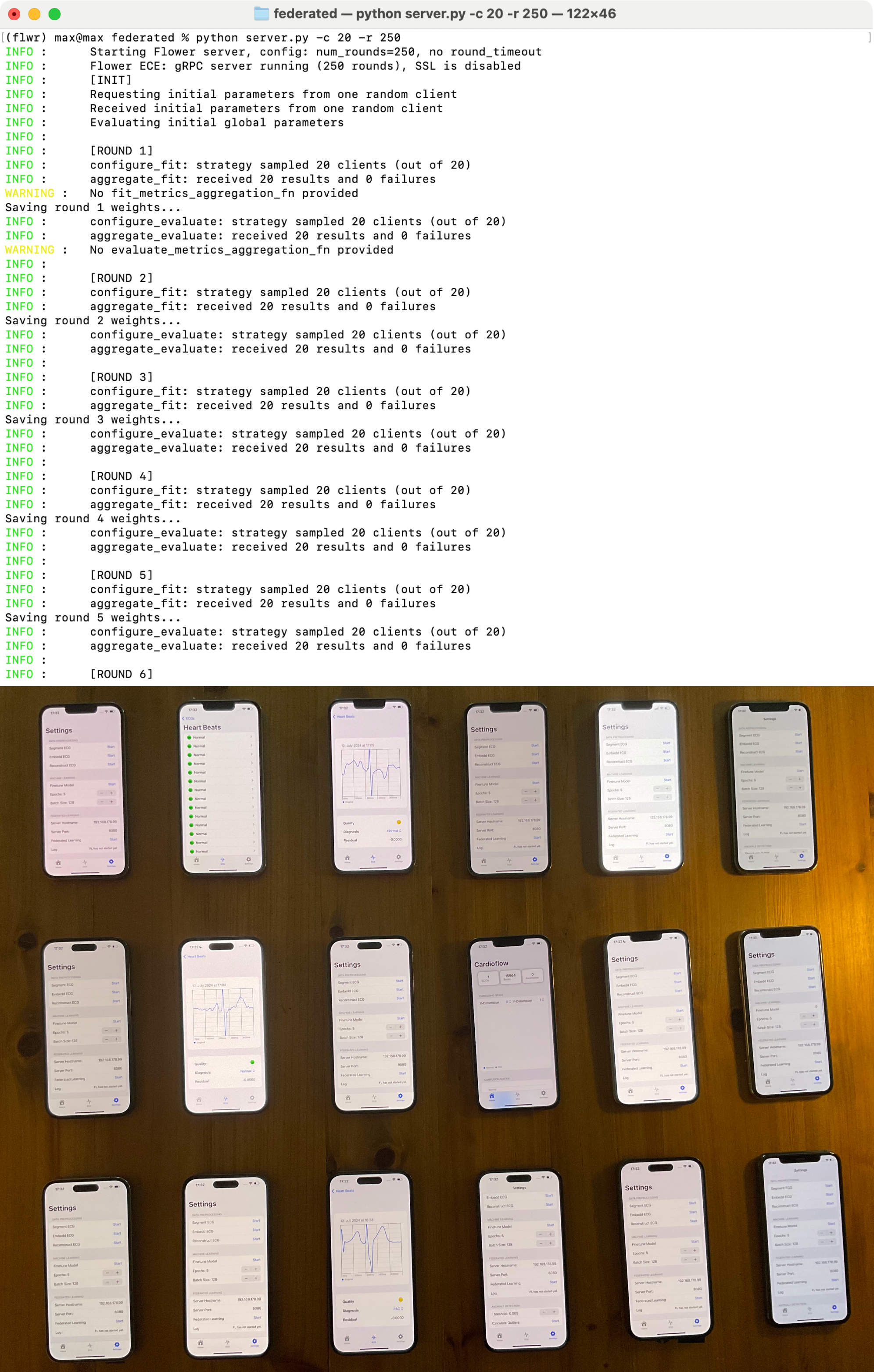

Supplement: S2 Fig — Depicts the study setting, including the devices (excluding tablets) and the console input and output of the server. (PNG) [file pdig.0000793.s002.png]

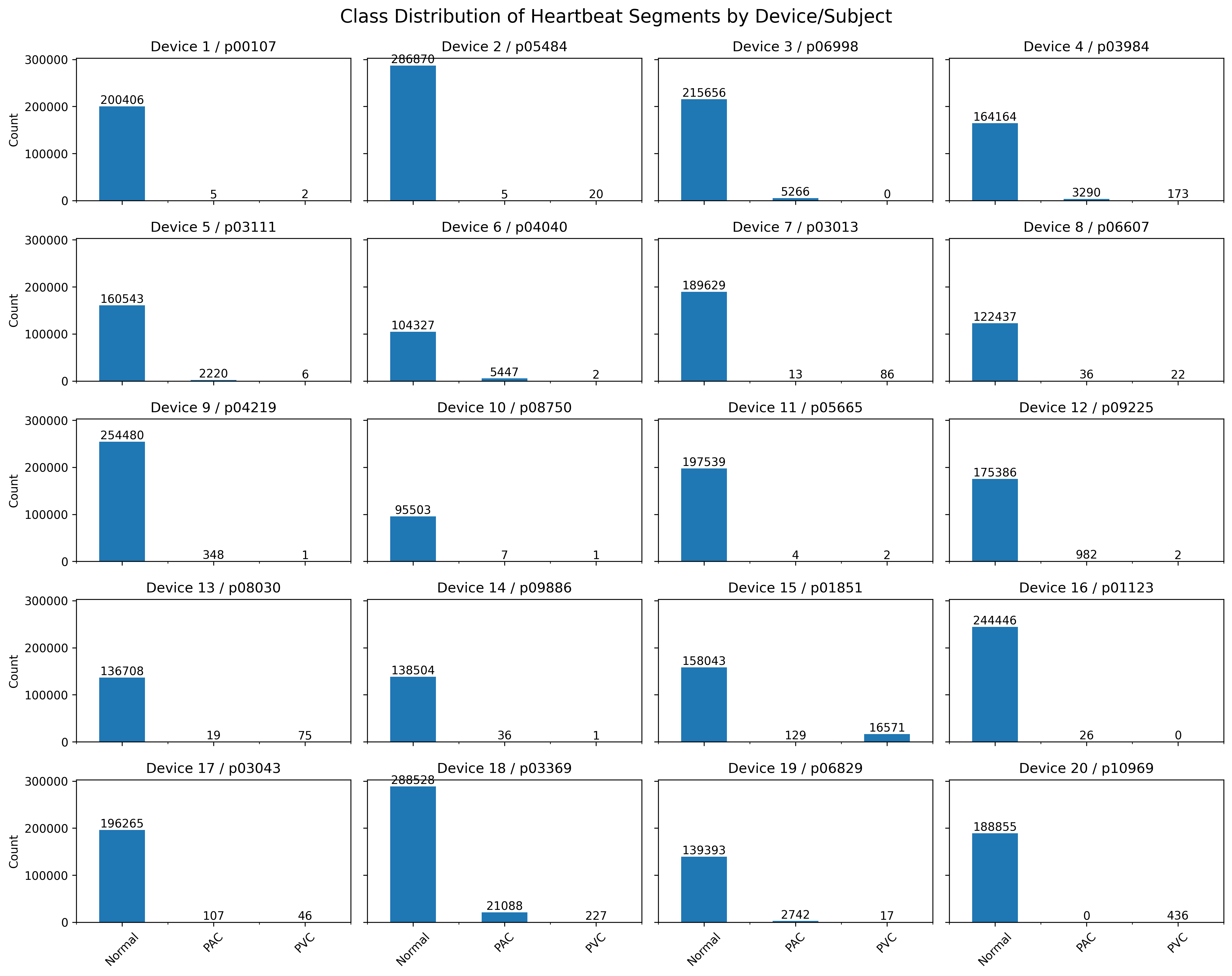

Supplement: S3 Fig — Histograms to display the distribution of classes across devices. (PNG) [file pdig.0000793.s003.png]

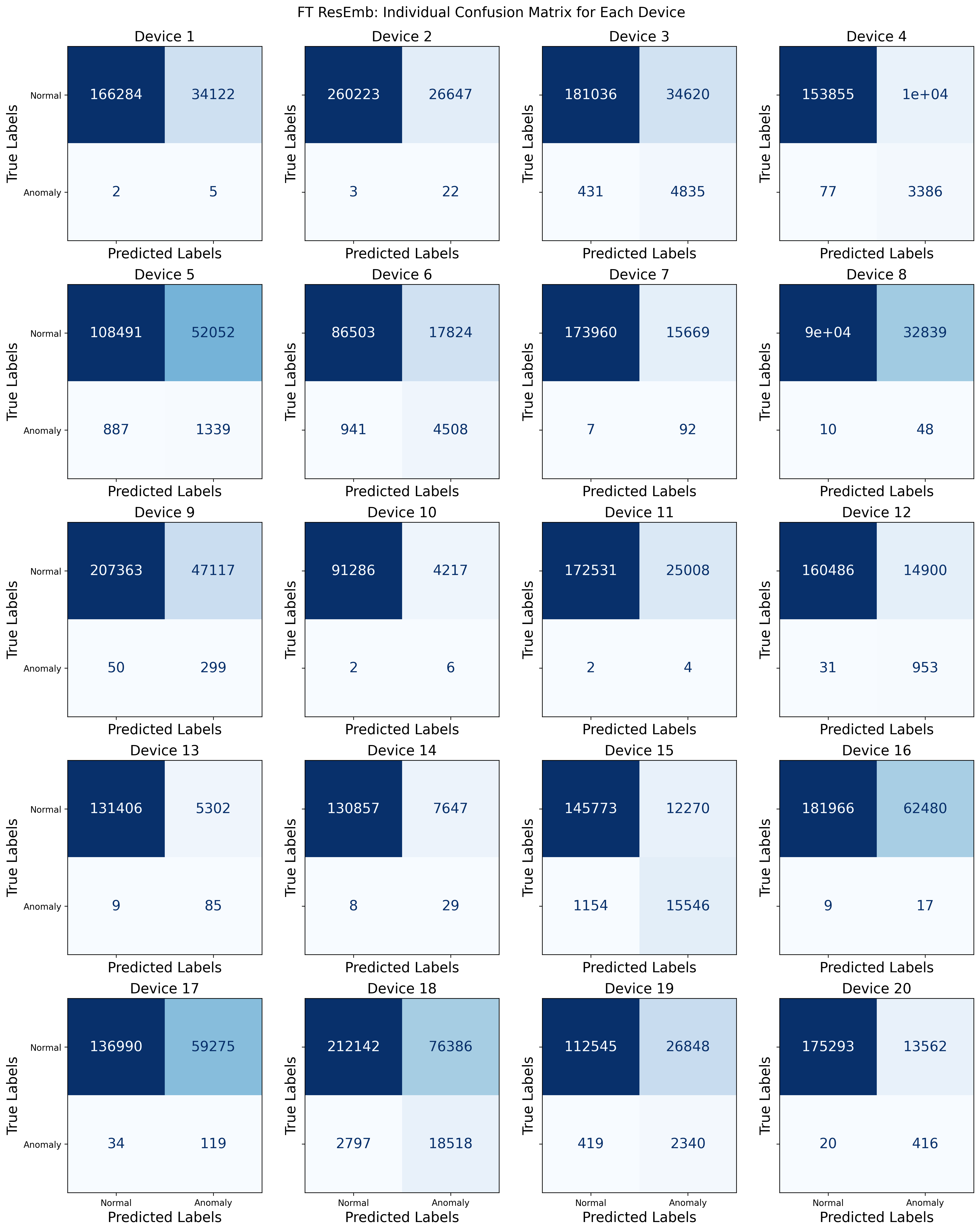

Supplement: S4 Fig — Confusion matrices to evaluate the performance of the individual devices. (PNG) [file pdig.0000793.s004.png]

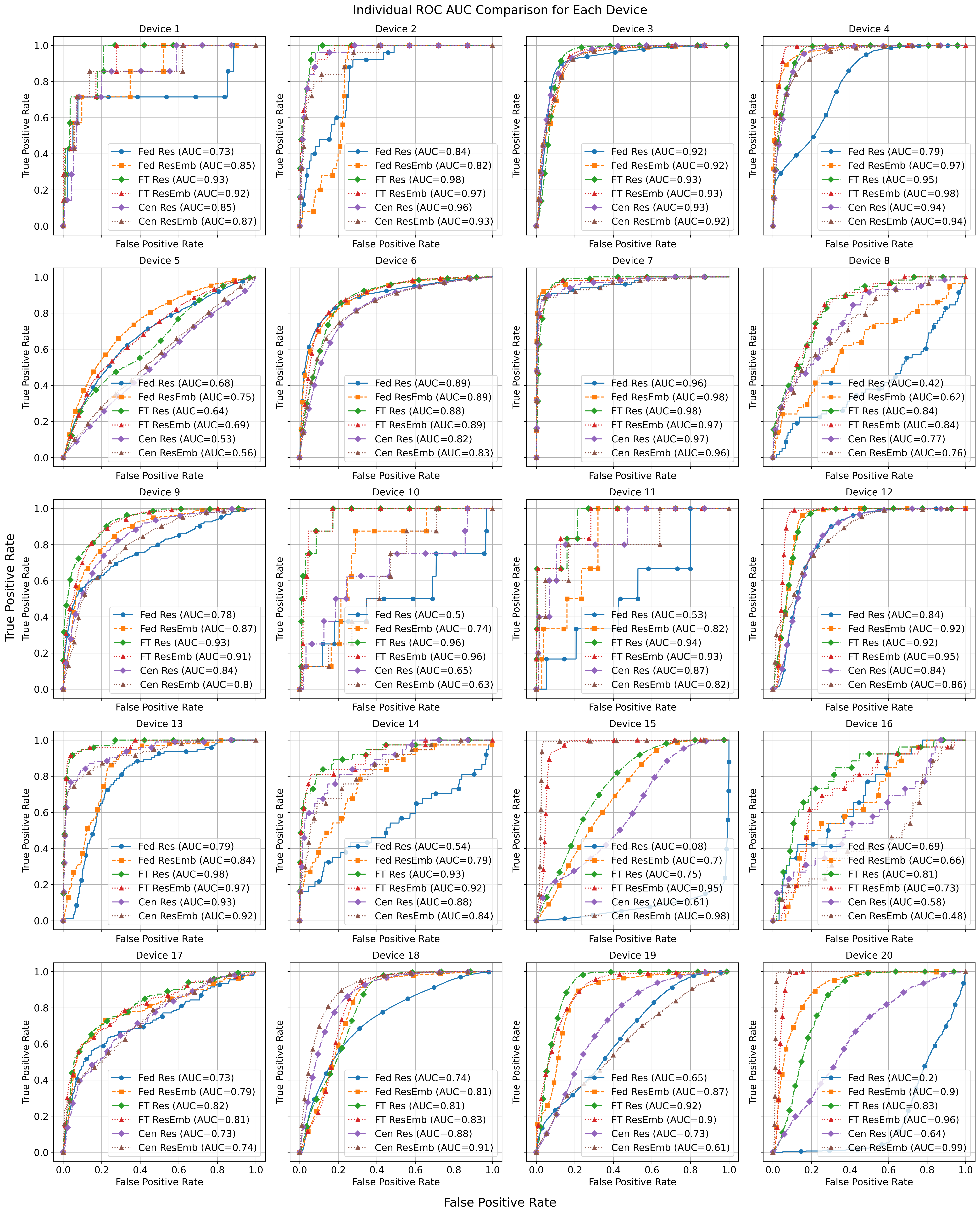

Supplement: S5 Fig — ROC curves to assess the performance of the approaches presented for each individual device. (PNG) [file pdig.0000793.s005.png]

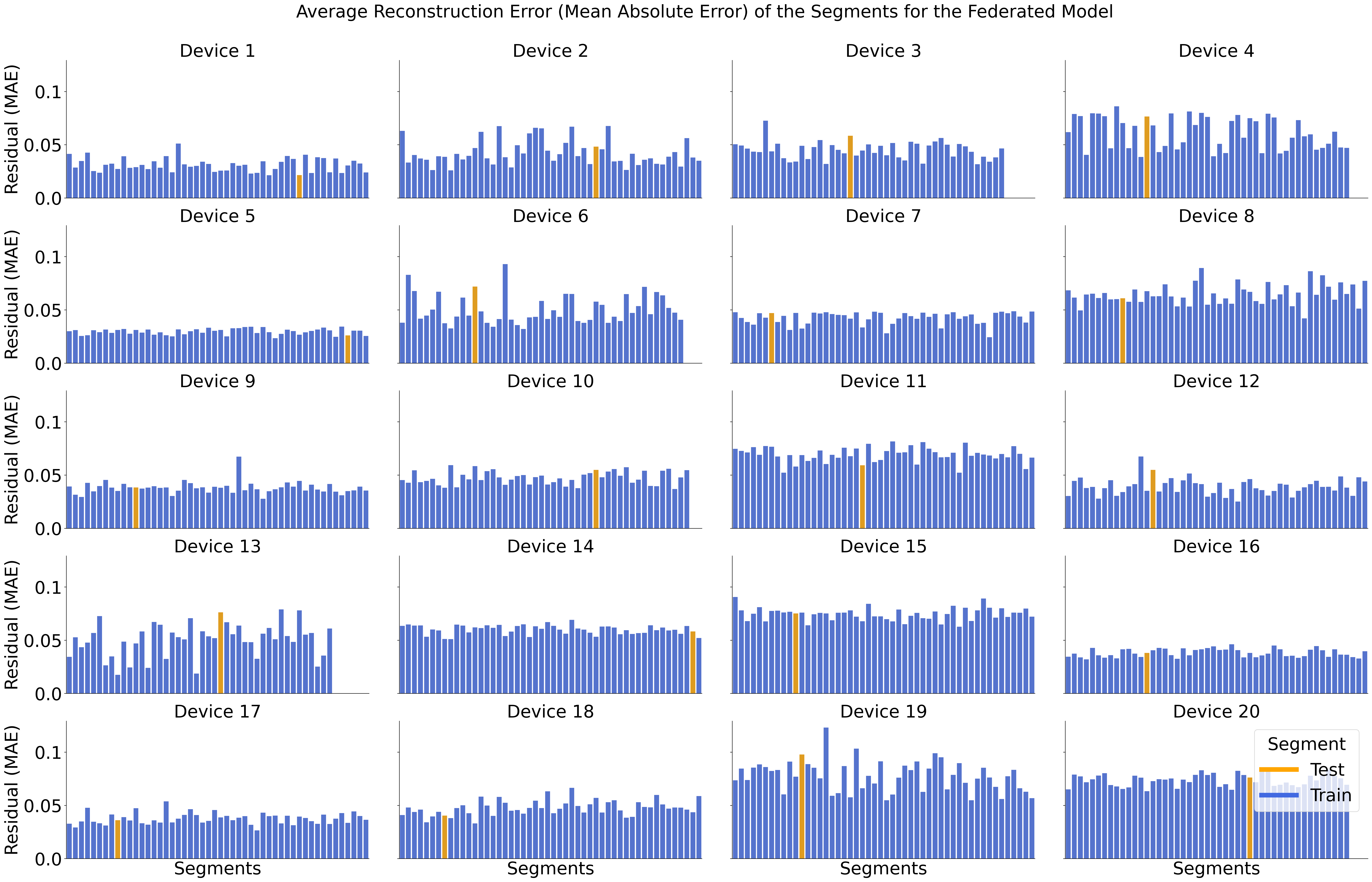

Supplement: S6 Fig — Loss per segment returned per device after training the federated model is presented, with the test segment highlighted to detect possible overfitting. (PNG) [file pdig.0000793.s006.png]

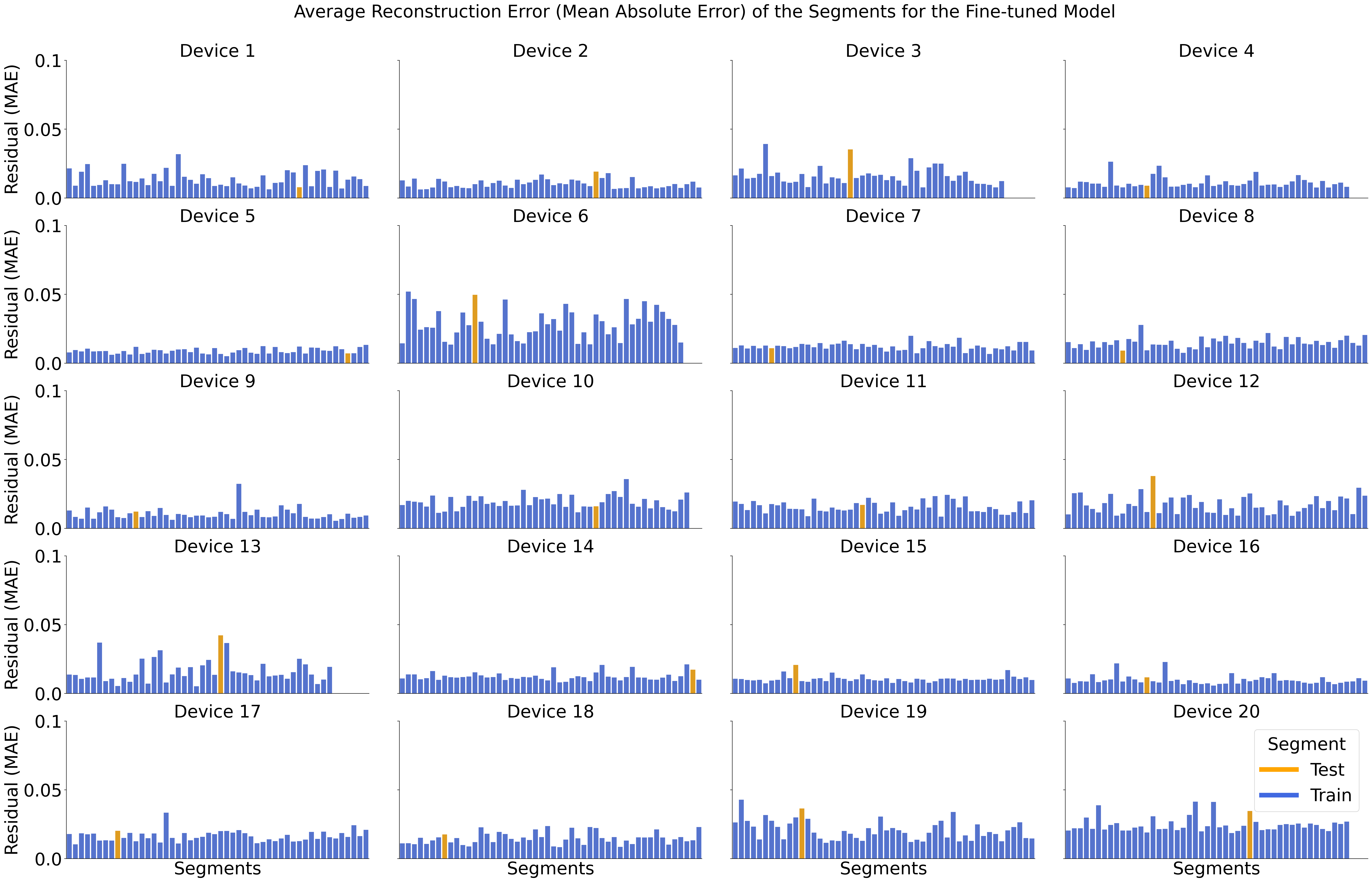

Supplement: S7 Fig — Loss per segment for each device after fine-tuning the model is shown, highlighting the test segment to identify possible overfitting. (PNG) [file pdig.0000793.s007.png]

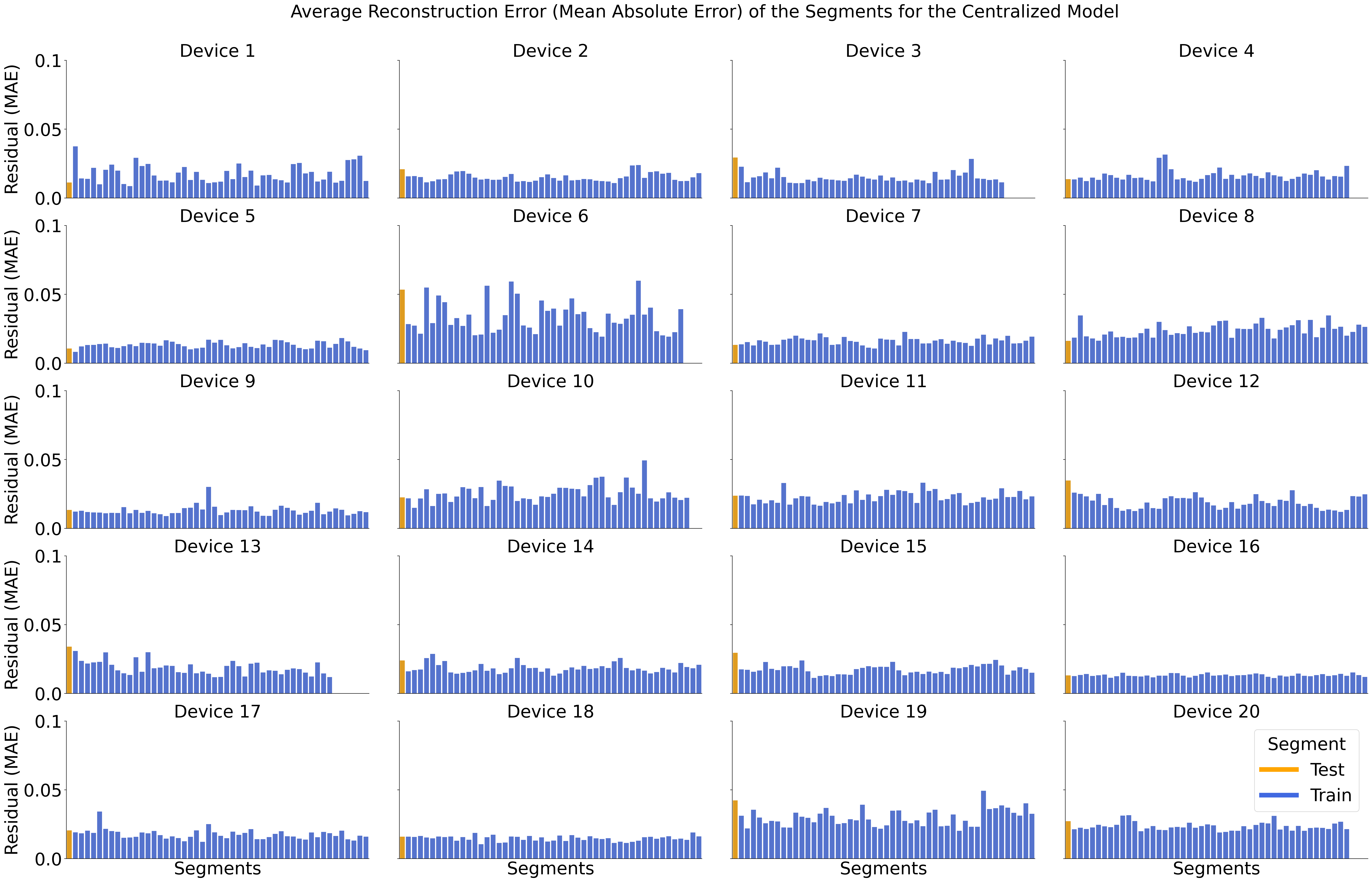

Supplement: S8 Fig — Loss per segment returned per device after training the centralized model is presented, highlighting the test segment to identify possible overfitting. (PNG) [file pdig.0000793.s008.png]

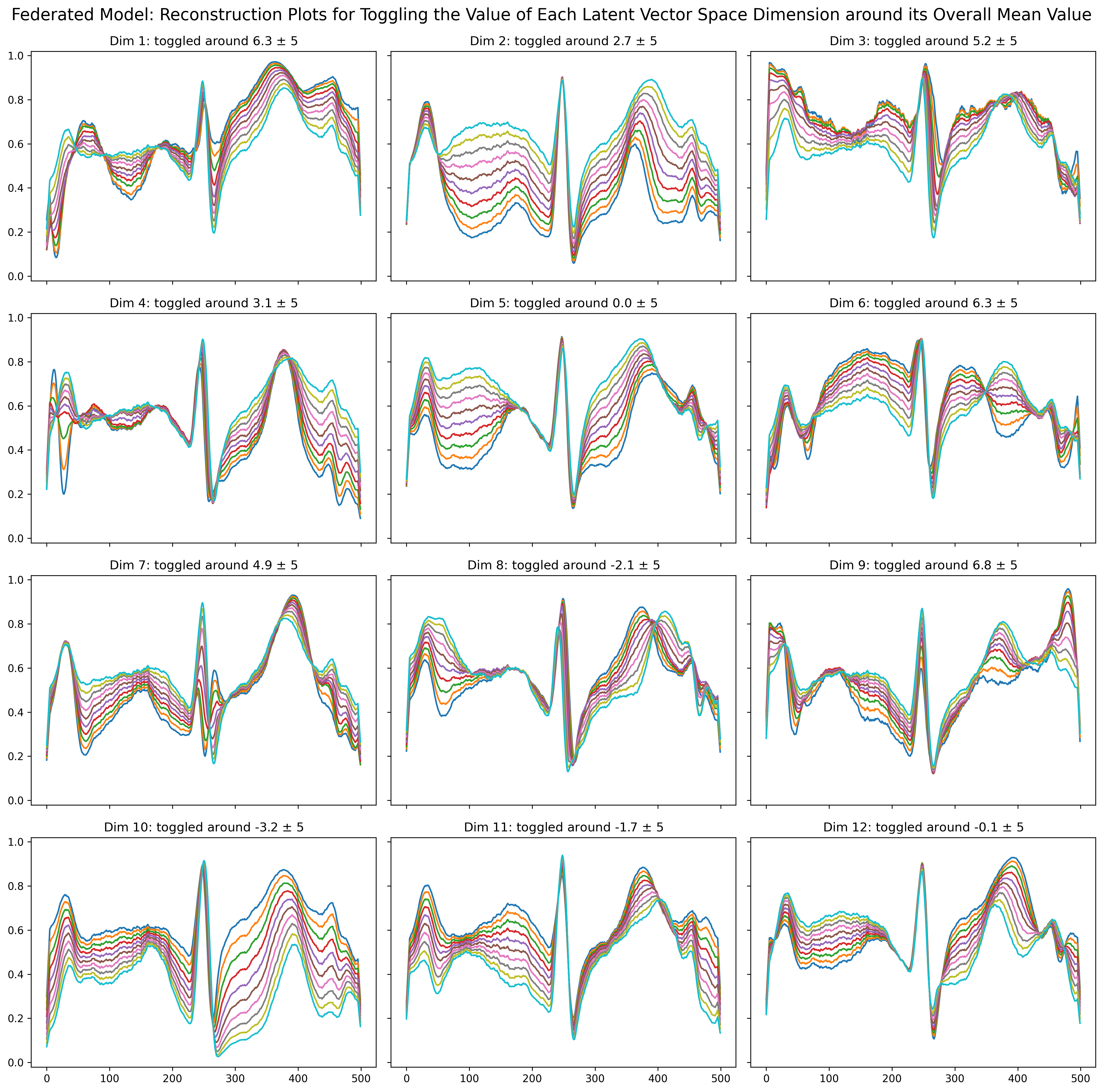

Supplement: S9 Fig — Reconstructed signals of the federated autoencoder are illustrated by varying individual dimensions of the latent vector space to test the model’s ability to generate realistic ECG signals. (PNG) [file pdig.0000793.s009.png]
