# Supplementary material for: Cross-device federated unsupervised learning for the detection of anomalies in single-lead electrocardiogram signals
Source: PLOS Digit Health. 2025 Apr 7;4(4):e0000793. doi: 10.1371/journal.pdig.0000793 (PMC11975069; doi:10.1371/journal.pdig.0000793)
Supplement: Attachment — Submitted filename: Response to Reviewers.pdf [file pdig.0000793.s010.pdf]

Cross-device federated unsupervised learning for the detection of anomalies in single-lead electrocardiogram signals

Response to Reviewer Comments

PDIG-D-24-00344

We thank the reviewers for their detailed comments, which have greatly helped to improve the manuscript. Below, we provide a point-by-point response to the comments.

Reviewer #1

☑ *In their manuscript "Cross-device federated unsupervised learning for the detection of anomalies in single-lead electrocardiogram signals", Maximilian Kapsecker and Stephan M. Jonas present the results of a machine learning algorithm trained in a federated manner on 20 mobile phones for detecting anomalies in ECG data.*

I think this is interesting work presented in a generally well-written text (with the help of AI tools, as disclosed by the authors) for which the source code has been published. My major points of criticizm are that I do not understand the practical relevance of this training approach and that the analysis of the results leave a few questions, this should be discussed in more detail. This, plus a few minor issues, lead to my recommendation of a major revision.

Dear Reviewer,

We sincerely appreciate your detailed and thoughtful comments, as well as your recognition of the interesting nature of our work and the attention to our effort in publishing all relevant sources for reproducing the results.

The practical relevance of our decentralized training approach is deeply rooted in addressing data privacy concerns. A significant amount of health-related data, such as electrocardiograms (ECGs), is stored on mobile devices like smartphones. These data hold immense potential for training stable and generalizable machine learning models due to their diversity, encompassing various age groups, pathologies, cultural backgrounds, and more. However, leveraging this data in a traditional machine learning setting is not straightforward: individuals must give consent, and even then, strict ethical guidelines, legal requirements, and privacy laws must be followed.

We understand your observation regarding the use of open-source data in this study, which does not inherently require a federated learning framework. This is a valid point, and we acknowledge that when data is openly available and unrestricted, federated learning may not be necessary. However, in this work, open-source data was used as a proof of concept to evaluate the feasibility of federated learning systems under controlled conditions. The results of this study serve as a foundation and motivation for future large-scale, patient-based studies involving diverse populations. Furthermore, the framework can be extended to less abundant physiological signals beyond ECGs and rare/different ECG pathologies, which may present even greater challenges for traditional machine learning approaches.

Federated learning enables us to overcome (some of) these challenges by allowing training across distributed data stored on devices while preserving privacy. This approach ensures that models benefit from the diverse range of data without compromising user privacy.

We have refined parts of the introduction to emphasize the motivation behind this work more clearly. Additionally, we have expanded the analysis of the results throughout the manuscript to address the points you raised in your review.

Thank you once again for your valuable feedback. Should you have any further questions or comments, we would be more than happy to address them to meet your expectations for publication.

☑ *Training algorithms for analyzing medical data in a federated (privacy-preserving) way and ideally without going through the work of annotating the data is certainly relevant. However, the scenario described here relies on a synchronous availability of federated clients for the initial training, which does not seem very realistic to me, at least not for the specific task here where a large dataset is already available and could be used to provide an initial model that is then fine-tuned locally and individually for each user.*

We agree that relying on the synchronous availability of federated clients is challenging in real-world scenarios due to issues like client dropouts and connectivity. However, our implementation with the Flower federated learning framework is designed to handle these situations. The framework allows training to proceed even if some clients are unavailable, as long as a minimum number of clients participate in each round to maintain privacy standards. If the number of active clients drops below this threshold, the training is paused until enough clients are available. Importantly, the framework is intended to function regardless of whether the application is actively open or running in the background.

Regarding the use of an open-source dataset, you are correct that this eliminates the immediate need for federated learning when training the base model. However, our goal was to simulate a realistic mobile scenario, using a dataset recorded in a mobile environment. The specific task – distinguishing normal heartbeats from premature contractions – was chosen due to the limited availability of open-source mobile-recorded ECG datasets. This proof of concept study highlights the potential of federated learning to leverage distributed ECG data on smartphones for more diverse tasks beyond the available open-source datasets.

As for the limitation requiring the CardioFlow application to remain open during training, we acknowledge that this is not ideal for practical use. While federated learning routines can technically run as a background process, current operating system restrictions prevent this for security reasons. We have noted this limitation and included plans in the future work section to address it by enabling background processing and improving client-handling mechanisms.

We have added these two concerns to our future work section and plan to address it in subsequent versions by enabling background processing and improved client-handling to enhance usability and practicality.

4.4 Limitations

The iOS ecosystem prohibits calling a function remotely when the application is closed, which makes unobtrusive and randomly scheduled FL as a background task currently infeasible. This limitation requires the predefined minimum number of user to be online and actively participating as a client node.

4.5 Future Work

Several limitations identified in this study provide a foundation for future efforts: (1) extending support to additional operating systems; (2) enabling federated learning routines to run as background processes, thereby enhancing usability without disrupting user activities; (3) implementing participation constraints based on device capabilities, operational state, and connectivity, as well as exploring client clustering techniques to optimize performance and resource utilization; and (4) repeating the experiment by iteratively sampling different subsets of the Icentia11k dataset and simulate an evolving number of clients over time to demonstrate robustness and generalizability, or conducting studies using a larger set of devices.

☑ *The evaluation of the prediction quality is based on single heartbeats, which is certainly a good basis and likely a deflation rather than an inflation of performance metrics. However, I would expect (I'm not an expert in this field, though) that one is actually interested in a per-patient prediction of premature contractions (or per-patient on a given day). A prediction quality assessment on such aggregated data could result in higher values for appropriate metrics if mis-classifications even out over multiple heartbeats.*

You're correct that while evaluating prediction quality based on single heartbeats provides a solid foundation, aggregating predictions on a per-patient or per-day basis can offer additional clinical significance. Excessive numbers of premature contractions (PCs), or beat pathologies in general, throughout the day can indicate serious health issues that require professional attention. For instance, increased frequencies of premature ventricular contractions (PVCs) have been linked to certain medical conditions, including cardiac arrhythmias and complications associated with infections like SARS-CoV-2. It must be noted however, that recording ECG in consumer-grade devices is often not performed regularly or over longer periods of time but mostly limited to few heartbeats per day or even week, for example, during exercises or a 30-second recording via a smartwatch.

In addition,patterns where PCs occur consecutively or might not be captured due to sparse recording, these patterns are not consistent and depend on underlying factors that are not known in advance. Therefore, relying on such methods could introduce inaccuracies.

In our study, the total count of PVCs over a day can be obtained by summing the single-event detections made by our model. This aggregation is a straightforward extension of our heartbeat-level predictions and provides the per-patient data that may be clinically relevant.

We acknowledge that our method's reliance on single-lead ECG data limits the detection to anomalies identifiable in a single heartbeat from the first lead. This includes certain forms of myocardial infarction, left and right bundle branch blocks, PCs, and first-degree AV block. We have added this consideration to the limitations section of our manuscript.

atrial fibrillation, which might be a more important topic for investigation. Also, some pathologies may not manifest in single heartbeats of the Goldberger lead I. Therefore,

☑ *Given that the present evaluation is based on a dataset distributed to the federated clients, a comparison to predictive performance after centralized training would be possible – and interesting to assess whether federation has a negative impact in this application.*

We agree that comparing the predictive performance of the federated model with that of a centrally trained model is important to assess any potential impact of federated learning in this application. Accordingly, we have trained a second model using centralized training on the

same dataset and parameters as the federated model. We assessed its performance metrics in the same manner to provide a direct comparison. We have updated the methodology, results, and discussion sections of the manuscript to include this comparison, highlighting the feasibility and accuracy of our approach.

2.4 Technical Setting

The same data, training and test distributions, and parameter settings were used to optimize the same model centrally on a single computer. The aim was to compare the centralized and federated models to determine the potential accuracy reduction caused by the federated setting. The centralized training was conducted on a Linux-based operating system with an *NVIDIA GeForce RTX 4090* for accelerated computing.

3.2 Anomaly Detection

Table 3. Anomaly detection metrics.

**Sample**	**Method**	**Threshold**	**Sensitivity**	**Specificity**	**Balanced Accuracy**
Overall	Fed Res	0.32	0.7654	0.7405	0.7529
Overall	Fed ResEmb	0.2603	0.7873	0.7246	0.7559
Overall	FT Res	0.1045	0.9506	0.6813	0.816
Overall	FT ResEmb	0.5838	0.866	0.8028	0.8344
Overall	Cen Res	0.1266	0.8933	0.695	0.7942
Overall	Cen ResEmb	0.4404	0.8181	0.7922	0.8052

The corresponding AUC are 0.82 for the federated model, 0.87 for the centralized model, and 0.89 for the fine-tuned model (cf. Fig 4).

Comparing *Fed ResEmb* to *Cen ResEmb*, the performance loss due to the federated setting was moderate, with reductions of 0.0308 in sensitivity, 0.0676 in specificity, and 0.0493 in balanced accuracy.

4.2 Anomaly Detection

impair the analysis. The performance of the centralized approach was slightly better, but did not yield a significant improvement over the results of the federated model.

☑ *If I understand correctly, the authors used 20 of 11,000 cases from a publicly available dataset. In the present scenario, it makes perfect sense to use one per client, and should not be viewed as using very little of the available data. However, this opens a good opportunity to assess how well the approach generalizes to other data by repeatedly sampling other 20 cases and obtaining confidence intervals for an overall performance metric. Also, it would be interesting to see how deterministic the results are for one set of 20 cases, i.e., to which extent performance differs between repeated runs even if the order of data used, accessible random seeds, etc. are fixed. (I'm more familiar with training deep neural networks on GPUs where this can be an issue; I'm not sure how deterministic the combination of federated training, mobile phones, the autoencoder architecture and the training scheme used here can be expected to be.) Combined with a federated-vs-centralized assessment, a centralized training on more than 20 clients could be used to estimate generalization in case more federated clients were involved.*

You are correct in noting that utilizing only 20 out of 11,000 cases, with one case per client, may raise concerns about the generalizability of our results. Ideally, repeatedly sampling different sets of 20 cases to obtain confidence intervals would provide a more robust

assessment of our model's performance across diverse patient populations. Similarly, evaluating the determinism of our results through multiple runs with fixed parameters would offer valuable insights into the consistency and reliability of our approach.

However, our current study faced limitations that constrained the ability to perform such extensive evaluations, since we explicitly decided to not simulate the federated learning devices but use physical devices as part of the feasibility evaluation:

1. Manual Data Handling: The process of transferring and loading subject data from the Icentia11k dataset onto each device required manual intervention. Additionally, establishing connections to the federated learning (FL) server and executing training routines were also manual processes. Automating these steps was beyond the scope of our prototype implementation, making the repetition of experiments with multiple subsets of 20 subjects (each subject is of size ~1 GB) highly time-consuming and resource-intensive.

2. Device Availability: The limited available time slots of the devices further restricted our capacity to conduct numerous training runs. Coordinating multiple sessions to accommodate different subsets of clients was not feasible within our project's timeframe.

Despite these constraints, we endeavored to ensure the robustness of our findings by:

Confidence Intervals: With the initial sample of 20 devices, we calculated confidence intervals for the AUCs for the different approaches using DeLong's method, demonstrating the statistical significance and superiority of the fine-tuning approach.

Centralized Model Training: In response to your earlier suggestion, we also trained a centralized model using the 20 samples. This comparison highlighted any potential discrepancies between federated and centralized training paradigms, reinforcing the validity of our federated approach.

Regarding the determinism of our results, we have ensured that our model architecture, training processes, and aggregation methods are designed to be deterministic when using fixed parameters. To the best of our knowledge, there are no inherent random or non-deterministic components in our implementation. Nevertheless, we acknowledge that variations in device hardware, such as CPU architectures and numerical precision, could introduce minor differences in outcomes – particularly when executed on different hardware. Therefore, we have acknowledged general system challenges in the limitation section.

To facilitate reproducibility and allow for independent verification of our results, we have made all source code, data handling procedures, and detailed manuals available in our GitHub repository https://github.com/CardioKit/cardioflow We encourage other researchers to replicate our study and explore further evaluations, such as repeated sampling and extended deterministic testing, to build upon our work.

Moving forward, we recognize the value of conducting more extensive experiments to thoroughly assess generalizability and determinism. Future iterations of our research will aim to automate data handling processes and optimize device coordination to enable such comprehensive evaluations.

We have updated the manuscript to reflect these points, including a discussion of the current limitations and our plans for future work to address them.

4.4 Limitations

limitation. In real-world applications, systems typically include a much larger number of devices, which can lead to complexities such as non-IID data and potentially lower accuracy. However, this larger scale can also contribute to the development of more robust and generalizable models – an aspect not addressed in this study.

4.5 Future Work

Several limitations identified in this study provide a foundation for future efforts: (1) extending support to additional operating systems; (2) enabling federated learning routines to run as background processes, thereby enhancing usability without disrupting user activities; (3) implementing participation constraints based on device capabilities, operational state, and connectivity, as well as exploring client clustering techniques to optimize performance and resource utilization; and (4) repeating experiments by iteratively sampling different subsets of the Icentia11k dataset to demonstrate robustness and generalizability, or conducting studies using a larger set of devices.

☑ *The limitations section briefly touches on using the presented approach in a real-world scenario. I would recommend elaboating on this a bit, as there will be further complicating factors like communication latency, clients dropping out (loss of network connection, loss of power), and others, which may introduce robustness requirements in addition to the requirements mentioned in Section 2.3.*

After reconsidering the limitations of our approach, we have expanded this section to provide a more comprehensive discussion. This revision includes aspects that currently hinder the robust execution of an autonomous overall system and addresses critical challenges associated with real-world deployment. The revised limitations read as follows:

This study was conducted using twenty mobile devices of varying generations, all operating on the iOS platform. While this provided a cross-device environment, it inherently limits the generalizability of our findings, especially considering Android's dominant global market share. Consequently, the exclusive focus on iPhone devices restricts the applicability of *Cardioflow* to a broader range of users and operating systems. Additionally, the relatively small scale of testing devices poses another limitation. In real-world applications, systems typically include a much larger and over the time evolving number of devices, which can lead to complexities such as non-IID data and potentially lower accuracy. However, this larger scale can also contribute to the development of more robust and generalizable models – an aspect not addressed in this study.

Deploying the proposed approach in real-world scenarios also presents several operational challenges. Factors like communication latency and the possibility of clients dropping out due to network instability or power loss can significantly impact system performance. Addressing these issues requires enhanced robustness measures beyond those discussed in Section 2.3. For example, implementing constraints to ensure that only devices with stable network connections, sufficient power, and minimal conflicting tasks participate in computations can help mitigate these challenges [11]. However, such constraints must be carefully designed to avoid introducing selection bias.

Detailed comments

- [x] *l 2: "mobile devices have evolved" is quite vague, is this about mobile phones having evolved to tools for multiple purposes, measurement devices having evolved to analysis capabilities?*

> We have clarified the statement to the effect that mobile devices refer to smartphones and tablets and specified what is meant by advancements.
>
> In recent years, advancements in mobile device hardware and software have transformed smartphones and tablets from simple communication tools into powerful computational platforms equipped with advanced sensors. These devices are now capable of recording, processing, and analyzing high-resolution biomedical signals directly on-device [1]. The

- [x] *l 56: I think "respectively" should be followed by a comma (but I'm not a native speaker)*

> Done.
> achieved an accuracy, precision, recall, and F1 score of 73.0%, 66.6%, 73.0%, and 69.7%, respectively, in the prediction of cardiac arrhythmias.

- [x] *l 112: the dataset itself should be cited here in addition to publications about the dataset*

> Done.
> Icentia11k is an open-source dataset of 11,000 subjects' ECG recordings [26,43–45]. The
>
> 43. Tan S, Ortiz-Gagné S, Beaudoin-Gagnon N, Fecteau P, Courville A, Bengio Y, et al.. Icentia11k Single Lead Continuous Raw Electrocardiogram Dataset (version 1.0);. https://doi.org/10.13026/kk0v-r952.
> 44. Tan S, Androz G, Ortiz-Gagné S, Chamseddine A, Fecteau P, Courville A, et al. Icentia11K: An Unsupervised Representation Learning Dataset for Arrhythmia Subtype Discovery. In: Computing in Cardiology Conference (CinC); 2021.

- [x] *l 113: I would find "3 to 14 days" easier to read.*

> Done.
> long-term recordings consist of single-lead ECG signals collected over 3 to 14 days and

- [x] *l 121: an explanation of "fifty segments" in terms of number of samples or time span covered would be helpful*

> Done. We specified:
> of subjects determined by the availability of mobile devices. The data for each subject consist of fifty segments, each representing approximately 70 minutes of ECG recording, resulting in a substantial total recording time. The segments were parsed into a

- [x] *l 240f: I would write the sentence as "The T-distributed stochastic neighbor (t-SNE) method was used …"*

> Done.
> the training and test segments and to detect possible overfitting. The t-distributed stochastic neighbor (t-SNE) method was used to reduce the data to a two-dimensional

☑ *Fig. 4: I would consider omitting this figure, it is not particularly helpful. The message of "20 clients" could instead be included in Fig 3, naming the clients 1, 2, 3, 20 and adding "…"*

We have removed Figure 4 from the manuscript and have adjusted the manuscript as suggested.

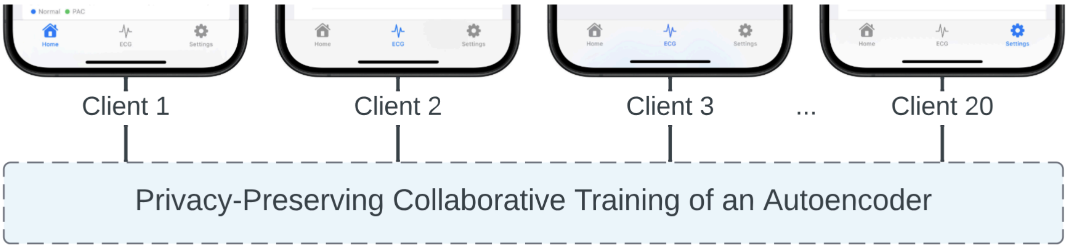

Fig 3. The main views of the Cardioflow application, including the home screen (Client

☑ *Fig. 5: Similarly, this screenshot of console output does not contain too many insights relative to the size of the figure. Maybe make this one a supplementary figure?*

We also agree on this point. We merged the study setting (previously Fig. 4) and the console output (previously Fig. 5) in one figure and made them available as supplementary elements.

S1 Fig. Study setting. Depicts the study setting, including the devices (excluding tablets) and the console input and output of the server.

☑ *l 250: should read "Euclidean" and not start a new paragraph after the formula*

Done.

is calculated as follows

$$a_k^{(i)} = d(v_k, \frac{1}{n}\sum_{j=1}^{n} v_j^{(i)})$$

where d is the Euclidean distance and n the total number embedded vectors of subject i.

☑ *Fig. 7: Here, I would recommend using different line types to make the figure greyscale-/colorblind-safe. (Same problem in Fig 6, but I doubt using different symbols would really help there.)*

Thank you for pointing out this important point. We have made sure that Fig. 7 is now grayscale and colorblind safe. We have also tried to optimize Fig. 6 in this respect. However, with a graphic like this, as you pointed out, there are limited ways to provide for colorblindness.

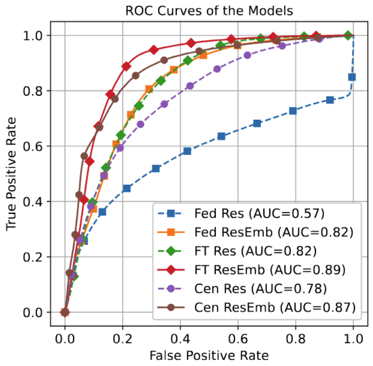

Fig 4. ROC curve and AUC comparison.

☑ *l 453: should read "proved feasible"*

Done.

Running the federated optimization process on twenty mobile devices in a real-world setting proved feasible and yielded high sensitivity and specificity for detecting

☑ *l 456: I think "proof of concept" should not be hyphenated here*

Done.

results are a proof of concept for federated unsupervised learning in a cross-device

☑ *Captions for supporting figures: I would rephrase the captions to omit "The figure shows"*

Done.

S2 Fig. Data Distribution. Histograms to display the distribution of classes across devices.

S3 Fig. Confusion Matrices. Confusion matrices to evaluate the performance of the individual devices.

S4 Fig. ROC Curves. ROC curves to assess the performance of the approaches presented for each individual device.

S5 Fig. Model Loss Federated. Loss per segment returned per device after training the federated model is presented, with the test segment highlighted to detect possible overfitting.

S6 Fig. Model Loss Personalized. Loss per segment for each device after fine-tuning the model is shown, highlighting the test segment to identify possible overfitting.

S7 Fig. Model Loss Personalized. Loss per segment returned per device after training the centralized model is presented, highlighting the test segment to identify possible overfitting.

S8 Fig. Reconstructions. Reconstructed signals of the federated autoencoder are illustrated by varying individual dimensions of the latent vector space to test the model's ability to generate realistic ECG signals.

☑ *l 481f: Unless the template text needs to be kept verbatim, I would use the applicable variant: "the authors … take …"*

> Done.
>
> readability and to eliminate typos and grammatical errors. Subsequently, the authors reviewed and edited the content as needed and take full responsibility for the content of the publication.

☑ *References: Should be double-checked for punctuation in URLs and arXiv identifiers, as well as capitalization of names in titles (e.g., Bayes) and journal names.*

> Thank you for bringing this issue to our attention. We encountered capitalization problems with several articles, including the "Auto-Encoding Variational Bayes" paper. We have updated the bibtex items and have thoroughly reviewed the remaining citations for consistency. To illustrate the specific case of the Bayes paper, we have included a screenshot showing the corrected reference below:
>
> 51. Kingma DP, Welling M. Auto-Encoding Variational Bayes; 2022. Available from: https://arxiv.org/abs/1312.6114.

☑ *Figure S2: The text is very small and hard to read.*

> We have enlarged the text, especially the numbers within the confusion matrices.

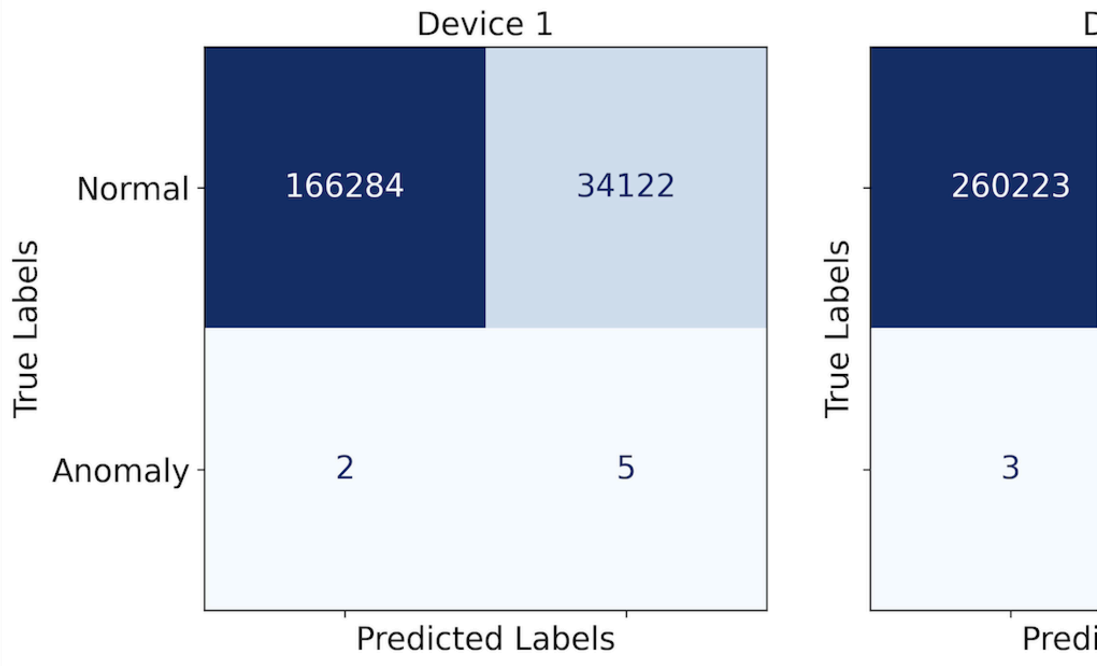

☑ *Figure S6: The variation is described only vaguely in the caption and in the text. I would appreciate some more detail on the range of variation.*

> We agree that additional information on the range of variation is necessary. Therefore, we have included the range of variation in the title of each subplot and added to the overall title

that the value varied by is the mean of the respective dimension.

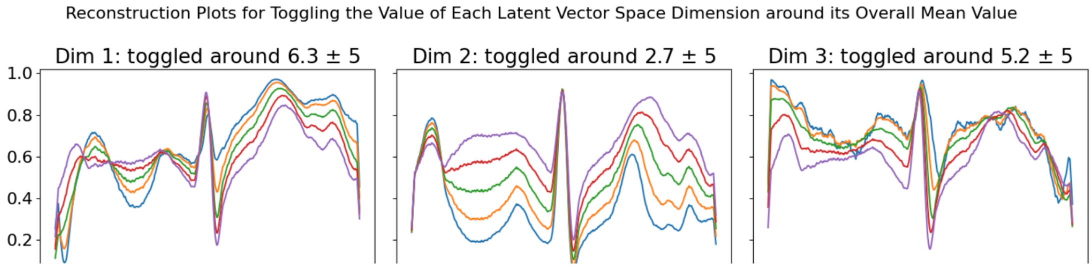

Reviewer #2

- ☑ *The manuscript presents a framework for the analysis of ECG signals from wearable devices, following a federated learning (FL) approach. The goal of the authors is to test the deployment of a machine learning autoencoder model within a GUI on mobile devices (iOS namely) which allow for analysis and semi-clinical output. The approach starts with a base model trained on open-source data and if self-supervised tuned on individual readings. The authors have a clear goal, nonetheless, their narrative has some gaps which need to be addressed before publication.*
  Also, I believe the focus of the manuscript should be "a model which is deployed as part of an app" but not focus as much the app per say – focus on the technology and not the vessel of it.
  Because of the inherent high value of the research, the manuscript should be classified as "Accepted with major revisions."
  Suggestion/needed additions are as follow per section:

> Dear Reviewer,
>
> We highly appreciate your thoughtful comments and thank you for recognizing the value of our research. After carefully reading your feedback, we believe it may be helpful to clarify an aspect of our approach that could have been misunderstood.
>
> It seems there might be a perception that the base model was trained centrally using the open-source Icentia11k dataset. While it is true that we utilized the Icentia11k dataset for base model optimization, this process was **not** conducted centrally. Instead, the base model was trained in a mobile environment using federated learning. This included sending model weights back and forth between the devices and the central server during the federated learning routine. Once this process concluded, the base model – already residing on the devices – was further fine-tuned locally on each device using the same data that had been involved in the federated setting. This fine-tuning occurred without sharing weights and focused solely on optimizing the model for the on-device data.
>
> This two-step approach was designed to mitigate potential effects caused by data from other subjects in the Icentia11k dataset that had been used on different devices during the federated learning process. If this clarification was already apparent, we kindly ask you to consider it redundant. Nonetheless, we have taken steps to sharpen these explanations in the revised manuscript to ensure this process is clear.
>
> Thank you again for your valuable input, which has greatly contributed to improving our work.

- ☑ *Author Summary*
  At the author summary, the authors mention:
  "mitigate the impact of data heterogeneity, we implemented on-device fine-tuning of the federated model for personalization"
  The section 2.5 titled "Evaluation" should have explain this method better and yet it did not. It is not clear how ground-truth data for validation is obtained.

> Thank you for your insightful comment.
>
> The ground-truth data for validation come from the Icentia11k dataset. We randomly selected data from 20 subjects within this dataset (11,000 subjects in total) and distributed each subject's data to a separate mobile device to simulate a real-world setting. This approach mirrors a scenario where individual devices collect data from distinct users, which is realistic

given that the Icentia11k dataset comprises recordings from wearable devices recorded in an actual study.

After training the base model in a federated fashion using the decentralized data stored on the twenty mobile devices, we performed on-device fine-tuning of the base model using each device's local data. This resulted in twenty fine-tuned models – one on each device. The purpose of this fine-tuning was to mitigate the impact of data heterogeneity by personalizing the global model to the specific data distribution of each device. We acknowledge that this was described too briefly in the "Technical framework" section and have expanded this accordingly:

> each round. With concluding the federated learning process, all devices shared the same model, which was referred to as the base model.
>
> To facilitate personalization, the base model was independently fine-tuned on each device for ten epochs using a batch size of 128. This personalization step is hypothesized to mitigate data heterogeneity and enhance accuracy by adapting to local data. To prevent overfitting to local data and preserve the model's generalizability – derived from training on a diverse range of participants – the duration of fine-tuning was carefully limited.

During the evaluation phase, we assessed and compared the performance metrics of all models, including the base model and the fine-tuned models – note that we included another ML model (this one was indeed trained centrally) for comparison purposes during the review process. The assessment is identical for all models included in the process. To make this more transparent, we have placed the following sentence at the beginning of the relevant paragraph in the evaluation section.

> The following computations were performed for all three results (i.e., residuals and embedding spaces) of the trained models, arising from either the FL routine (Fed), the fine-tuning process (FT), or the centralized (Cen) model optimization. The residuals

For a better overview of the models involved in the evaluation, we have added a table with a model overview (see response to your last comment).

☑ *Introduction:*
Grammar suggestions:
Line 99, change question to: How feasible is autoencoders implementation for federated unsupervised learning, in a cross-device real-world scenario?
Line 101 correct to: What is the performance of autoencoders in the detection of electrocardiogram anomalies as part of a cross-device federated unsupervised environment?
Line 103 correct to: Can on-device fine-tuning be sufficient for personalization and detecting baseline changes, thus addressing the challenge of non-IID data

Thank you for your suggestions, which in our opinion represent a more elegant way of formulating the research questions. We have therefore adopted your wording. In response to another reviewer's comment, we have added a short identifier at the beginning of each

research question to clearly associate it with the corresponding section in the discussion.

RQ1 Feasibility: How feasible is autoencoders implementation for federated unsupervised learning, in a cross-device real-world scenario?

RQ2 Anomaly Detection: What is the performance of autoencoders in the detection of electrocardiogram anomalies as part of a cross-device federated unsupervised environment?

RQ3 Inter-subject variations: Can on-device fine-tuning be sufficient for personalization and detecting baseline changes, thus addressing the challenge of non-IID data?

☑ *Materials and Methods*
Regarding data, the use of open source dataset Icentia11k is clear. Nonetheless, if all the dataset was used for training or not, is unclear. Further, the data loaded into the mobile apps is unclear regarding its origin – are these datapoints also part of the open source dataset, or were these newly generated by users? If the later is true, is there an ethic approval?

The Icentia11k open-source dataset was the only material used in the study. We acknowledge that this is not made clear enough and one gets the impression that other data, e.g. recorded by the Polar H10 - which would then certainly require ethical approval - is also used. We have reworded the potentially misleading parts of the article to make this fact clearer. The data of the twenty subjects of the 11000 subjects were randomly sampled (using a standard seed – 42 – to redraw the same samples for reproduction). Thus, in line with the maximal availability of mobile devices (twenty in total), only a small subset of the icentia11k dataset was chosen – each subject has a total of ~58 hours of ECG recording. Changes include:

1. Phrasing an outline in the introduction section to emphasize that the source data is open-source:

As part of this proof-of-concept study, a publicly available, mobile-recorded ECG data set was used and the data transferred to smartphones. A custom developed mobile application enabled privacy-preserving, unsupervised learning routines on this data and supported personalization by allowing fine-tuning of a federated trained model on local data, thereby accounting for non-IID effects.

2. The references to the possibility of recording ECGs with commercially available portable devices such as the Polar H10 have been removed so as not to give the impression that a data source other than Icentia11k, e.g. self-recorded data, was used.

FR1 The application must be able to ~~record ECG data directly from the Polar H10 device, collect existing ECG data from the device's health application, and upload~~ receive an ECG signal – in a common format such as a comma-separated values file – including the option to provide annotations for visualization purposes. The collected data must be stored in an application-internal memory.

3. Explicit reference to patient-based studies being part of future work, in which data sources other than the Icentia11k, e.g. Polar H10 recordings, could be used.

Looking ahead to future work involving a patient-based study, it is also possible to record an ECG using the Polar H10 device as a data source.

☑ *The authors also miss the explanation of why they target development into the iOS platform and used the Polar H10 as measurement device (again, it misleading as while a measuring device is listed, no human-lead experiments are mentioned). Several wearables, of different brand provide ECG recording, analysis and export. Thus, the sole deployment on apple devices and one sole type of wearable seems short at best. Thus, the authors are asked to either run experiments for the android ecosystem and also use different wearables or given better explanations regarding their decisions (materials and methods section).*
Thus, the authors must at least provide:
- *Explanations regarding the sole targeting of iOS*
- *Explanations of the sole use of Polar H10 device*
- *Cohort of human testers (particularly important since the open-source dataset is centered on third-age population)*

The mention of the Polar H10 measurement device serves solely to motivate and enable future research for conducting studies in real-world assessment scenarios. In alignment with our revisions with reference to your previous comment, where we clarified that only open-source data were used within the scope of this study, references to the Polar H10 device have been shifted to the future work section to emphasize its role as part of potential future research endeavors.

The authors believe that in a research context, a prototyping-first strategy is the most practical approach. Attempting to accommodate all possible commercial devices within the initial implementation is usually infeasible unless a unified interface allows for a one-implementation-fits-all strategy. While cross-platform tools like Flutter or ReactNative exist, these do not currently support federated learning. Therefore, we decided to support only one operating system for this feasibility study. In addition, the iOS ecosystem is more homogeneous compared to the devices that run on Android. Therefore, iOS is a suitable candidate for prototyping, covering a variety of devices with a single implementation. Since Flower (the used FL library) has libraries for both iOS and Android, it is theoretically possible to use a mixed-device setup, which, however, would not influence the results as the underlying experiment is independent of the mobile operating system. In our context, a variety of capacities (CPU, battery, RAM) of the individual devices was more important, which we achieved by including devices of different generation.
Nevertheless, we acknowledge your concerns, particularly for follow-up implementations, and have extended the limitations section to address these points. First, implementing the prototype for a single platform, even when intended purely for research purposes, could introduce biases. For instance, statistical data indicate that iOS users generally have a higher household income compared to Android users. Second, the open-source dataset, which primarily represents a third-age population, limits the generalizability of the approach to younger demographics. To address this, we have expanded the limitations section accordingly and included additional demographic data to provide more context when introducing the dataset.

2.3 Mobile Application

Because iOS devices maintain a relatively homogeneous environment across different device generations and hardware capacities, this platform serves as a suitable basis for the prototype implementation. Since Flower also provides an Android implementation, extending the approach to a mixed-device setup remains a feasible direction for future work.

4.4 Limitations

This study was conducted using twenty mobile devices of varying generations, all operating on the iOS platform. While this provided a cross-device environment, it inherently limits the generalizability of our findings, especially considering Android's dominant global market share. Consequently, the exclusive focus on iPhone devices restricts the applicability of *Cardioflow* to a broader range of users and operating systems. Additionally, the relatively small scale of testing devices poses another limitation. In real-world applications, systems typically include a much larger and over the time evolving number of devices, which can lead to complexities such as non-IID data and potentially lower accuracy. However, this larger scale can also contribute to the development of more robust and generalizable models – an aspect not addressed in this study.

[...]

The demographic composition of the dataset, particularly the overrepresentation of participants in the later stages of life, present a limitation to the generalizability of the findings.

4.5 Future Work

Several limitations identified in this study provide a foundation for future efforts: (1) extending support to additional operating systems; (2) enabling federated learning

☑ *It is missing how the autoencoder was trained, namely hardware. Is it also missing how the model was adapted to run in CoreML.*
Regarding the federated learning pipeline, little is mention about tis implementation and if each individual refined model (in their respective devices) is then fetch back to the central server for model merging (whole purpose of federated learning – gathering data within the models without having access to the data per say).

As indicated in our first response, the autoencoder was solely trained on the mobile devices: (1) in a federated fashion; (2) through fine-tuning. The specification of the devices' hardware was provided in section **2.4 Technical Setting**.

To create the CoreML model, we first designed the architecture in PyTorch and then converted it into the CoreML format using "coremltools". For reference:
https://github.com/CardioKit/cardioflow/blob/main/src/model/generate_model.py
We have added a sentence to explain this matter:

2). The autoencoder architecture was developed using PyTorch and subsequently converted to CoreML format with coremltools[2].

Regarding the details on the implementation of the federated learning framework: We used an existing implementation of a (mobile) federated learning framework (flower), which was created and published in earlier research efforts. We acknowledge that this is currently nor clear.

realistic cross-device FL setting. The target platform was iOS, mainly using the Swift implementation of the *Flower* framework [47,48], which provides a library for federated learning routines.

We hope that with our previous answers and revisions, it is now clearer that the base model

was trained in a federated fashion - so weights were exchanged back and forth at this stage - and fine tuning was then done solely on the device, with no communication dependencies.

☑ *Results:*
In section 3.1. the authors miss on mention what Modern UI libraries were used for visualization and why.

Thank you for highlighting this miss. We have referenced the UI libraries, stating an author's definition of modern UI libraries.

straightforward. The integrated SwiftUI[3] library has a declarative style, which allowed for effective visualization of results and metrics, but further consideration is needed when presenting to non-expert users, such as in the case of a clinical trial.

☑ *There should be a section for results on the base model itself. Since you took an open source dataset, and used a costume autoencoder, then, validation metrics should have been mentioned and not only metrics after deployment in the devices. A common 70-30% training-validation data split is suggested.*
The base model can then be compared to the output after fine tunning. There should also be a comprehensive description of the fine-tune results (as text, not only figures).
The models acronyms need better explaining as well.

As mentioned above, the model training was conducted solely on-device. To clarify, the results of the base models (**Fed Res** and **Fed ResEmb**) and their fine-tuned counterparts (**FT Res** and **FT ResEmb**) are presented in Table 3 and the losses of the autoencoder training is illustrated in Fig S4/Fig S5. Additionally, following another reviewer's suggestion, we trained the same autoencoder in a centralized manner using the identical dataset for comparison, with these centralized results also included in Table 3.
We recognize the need for a more detailed description of the (fine-tuning) results and added additional numbers and expanded on including the relevant numbers for this research:

3.2.1 Generalized Approach

Using the reconstruction error in combination with the distance to the center of mass (ResEmb) as a predictor for anomalies resulted in the best AUC values. The corresponding AUC are 0.816 (95 % CI: [0.8148, 0.8176]) for the federated model, 0.872 (95 % CI: [0.8708, 0.8735]) for the centralized model, and 0.888 (95 % CI: [0.8873, 0.8892]) for the fine-tuned model (cf. Fig 4). The hypothesis that there is no statistical difference between the AUCs was rejected at a significance level of $p = 0.01$. At a threshold of 0.58, the fine-tuned model achieves a sensitivity of 0.87, a specificity of 0.80, and a balanced accuracy of 83% when PAC and PVC are combined into a single anomaly class. However, there are a large number of false positives that are likely caused by the number of normal ECGs of *barely acceptable* quality. The total number of false positives ($n = 721,185$) with *barely acceptable* signal quality was $424,337$. By excluding samples with *barely acceptable* signal quality, the best-performing approach, FT ResEmb, increased its AUC to 0.91 and demonstrated improvements of 0.0303 in sensitivity, 0.0482 in specificity, and 0.0393 in balanced accuracy.

Examination of the detection performance of the two pathologies shows strong discriminatory power for PVC, with $16,528$ of $17,109$ samples correctly identified, corresponding to a sensitivity of 0.9672. For PAC cases, $26,994$ of $34,382$ samples were correctly identified, corresponding to a sensitivity of 0.8231. The specificity remained unchanged as the above threshold calculated for all samples was used.

Comparing *Fed ResEmb* to *Cen ResEmb*, the performance loss due to the federated setting was moderate, with reductions of 0.0308 in sensitivity, 0.0676 in specificity, and 0.0493 in balanced accuracy.

3.2.2 Individual Approach

When determining a per-subject threshold for the fine-tuned model, the AUC value increased to above 0.9 in 14 cases, remained in the range of 0.8 to 0.9 in 4 cases, and was below 0.8 in 2 cases. Subject *p03111* demonstrated notably poor performance, particularly in segments 34 and 49. Upon closer examination and visualization, the signals in these segments exhibited non-smooth slopes and a low dynamic range. Accumulating the confusion matrices into one yielded $3,078,588$ true-negative, $52,567$ true-positive, $579,094$ false-positive, and $6,893$ false-negative results. These figures correspond to a sensitivity of 0.8841 and a specificity of 0.8417. Detailed subject-related results such as the confusion matrices (S3 Fig), ROC curves (S4 Fig) and the model loss (S5 Fig, S6 Fig, S7 Fig) were included as supplementary material. The reconstruction quality of the federated model is compiled in S8 Fig.

While we have not elaborated on the numbers and figures presented in the supplemental material within the main text, we have referenced them to enable interested readers to gain additional insights into the results beyond the core research questions.

Furthermore, we have improved the explanation of model acronyms and added an overview in the Evaluation section to introduce the different models and their respective purposes.

Table 2. Autoencoder model variants for the evaluation of the ECG anomaly detection.

**Identifier**	**Setting**	**Description**
Fed Res	Federated	Base autoencoder trained on data distributed to 20 devices. Anomalies identified using reconstruction residuals.
Fed ResEmb	Federated	Base autoencoder trained on data distributed to 20 devices. Anomalies identified using residuals and embedding vectors.
FT Res	Fine-tuned	Fine-tuned the base model locally on each device to adapt to individual data distributions. Anomalies inferred using residuals.
FT ResEmb	Fine-tuned	Fine-tuned the base model locally on each device. Anomalies inferred using residuals and embedding vectors.
Cen Res	Centralized	Autoencoder trained on centrally stored data. Anomalies identified using residuals.
Cen ResEmb	Centralized	Autoencoder trained on centrally stored data. Anomalies identified using residuals and embedding vectors.

Reviewer #3

- [x] *I have reviewed the manuscript titled "Cross-device Federated Unsupervised Learning for the Detection of Anomalies in Single-lead Electrocardiogram Signals." The authors present a novel idea of heartbeat anomaly detection using auto-encoders in a real-world scenario involving 20 mobile phones.*

  Strengths:
  1- The manuscript addresses a critical aspect of current clinical problems in cardiology, highlighting its significant scientific value.
  2- The proposed method of using federated unsupervised learning for anomaly detection in ECG signals is innovative and demonstrates practical applicability.
  3- Conducting experiments on 20 mobile phones showcases the feasibility of the approach in real-world scenarios, which enhances the practical relevance of the study.

> Dear Reviewer,
>
> We sincerely appreciate your thoughtful comments and your recognition of the strengths of our research. Your emphasis on its practical relevance and potential impact is especially encouraging. We remain fully open to addressing any additional concerns or suggestions you may have.

- [x] *Suggestions for Improvement:*
  1- Effect of Number of Clients: It would be interesting to see the effect of the number of clients on the final result. I suggest the authors run the same experiments with different numbers of clients (e.g., 4, 8, 16, 20) and report how the model's performance changes. This would provide a deeper understanding of the scalability and robustness of the proposed method.

> The authors agree that examining the impact of varying the number of clients on model performance is an intriguing and valuable direction for analysis. Such experiments would ideally strengthen the assessment of scalability and robustness of the proposed method.
>
> However, as outlined in a previous response, our study faced practical constraints that made such extensive evaluations unfeasible. Specifically, the limited availability of devices restricted our capacity to conduct multiple training runs with different client configurations. Coordinating additional sessions to vary the number of clients was not feasible within the project’s timeframe.
>
> Despite these limitations, we have taken alternative steps to ensure the robustness of our findings. We extended the comparison of the federated learning approach to a centralized training approach, assessing the performance and potential impact of the federated setting on model accuracy. This also addresses your second point for improvement.
>
> While we were unable to investigate the effect of varying client numbers in this study, we acknowledge its importance and have included it yet as a limitation as well as a key aspect for future research.
>
> We hope that this extended comparison offers an interesting and meaningful dimension for analysis. We greatly appreciate your understanding of the practical limitations faced in this study and thank you once again for your thoughtful suggestions, which have significantly informed our future research directions.

4.4 Limitations

limitation. In real-world applications, systems typically include a much larger number of devices, which can lead to complexities such as non-IID data and potentially lower accuracy. However, this larger scale can also contribute to the development of more robust and generalizable models – an aspect not addressed in this study.

4.5 Future Work

Several limitations identified in this study provide a foundation for future efforts: (1) extending support to additional operating systems; (2) enabling federated learning routines to run as background processes, thereby enhancing usability without disrupting user activities; (3) implementing participation constraints based on device capabilities, operational state, and connectivity, as well as exploring client clustering techniques to optimize performance and resource utilization; and (4) repeating experiments by iteratively sampling different subsets of the Icentia11k dataset to demonstrate robustness and generalizability, or conducting studies using a larger set of devices.

☑ *2- Comparison with Centralized and Local Learning: As the data is publicly available, it would be valuable to compare the results of centralized learning (all data in one place, without federated learning) and local learning (each client is trained and evaluated based on its data) against the federated learning approach already included in the study. This comparison would offer insights into the relative advantages and potential limitations of each method.*

This is a valid point as well. We already addressed this aspect in our previous response to your first suggestion for improvement. The analysis for comparison towards a centralized model has been conducted and incorporated into the manuscript at the following locations:

2.4 Technical Setting

The same data, training and test distributions, and parameter settings were used to optimize the same model centrally on a single computer. The aim was to compare the centralized and federated models to determine the potential accuracy reduction caused by the federated setting. The centralized training was conducted on a Linux-based operating system with an *NVIDIA GeForce RTX 4090* for accelerated computing.

3.2 Anomaly Detection

Table 3. Anomaly detection metrics.

Sample	Method	Threshold	Sensitivity	Specificity	Balanced Accuracy
Overall	Fed Res	0.32	0.7654	0.7405	0.7529
Overall	Fed ResEmb	0.2603	0.7873	0.7246	0.7559
Overall	FT Res	0.1045	0.9506	0.6813	0.816
Overall	FT ResEmb	0.5838	0.866	0.8028	0.8344
Overall	Cen Res	0.1266	0.8933	0.695	0.7942
Overall	Cen ResEmb	0.4404	0.8181	0.7922	0.8052

The corresponding AUC are 0.82 for the federated model, 0.87 for the centralized model, and 0.89 for the fine-tuned model (cf. Fig 4).

Comparing *Fed ResEmb* to *Cen ResEmb*, the performance loss due to the federated setting was moderate, with reductions of 0.0308 in sensitivity, 0.0676 in specificity, and 0.0493 in balanced accuracy.

4.2 Anomaly Detection

impair the analysis. The performance of the centralized approach was slightly better, but did not yield a significant improvement over the results of the federated model.

Thank you for your suggestion regarding training a separate model for each participant from scratch. We acknowledge that this approach could lead to the model overfitting to an individual's data distribution. While it might work well for tracking changes over time within a single person's ECG – since deviations from the strongly learned data distribution would be readily detectable – the absence of a diverse range of ECG data could make the system prone to significant errors. For example, if a person primarily exhibits pathological ECGs, training a model solely on this data would result in the model learning the pathology as normal, without the opportunity to compare it against a broader, healthier population.

We have refrained from reporting the "local only" autoencoder training in the manuscript, as training on only a fraction (1/20th) of the subset and comparing the results to the full subset might be confusing for the readers and a direct comparison might not yield additional insights.

We had similar considerations when fine-tuning the model on individual data. Given the established weights of the base model, changes during fine-tuning were not overly volatile. However, we carefully limited the fine-tuning to ten epochs (instead of a higher number like 50) to prevent overfitting to the individual's ECG data distribution, ensuring that the model could still identify pathologies. We have described this more thoroughly in the revised manuscript.

To facilitate personalization, the base model was independently fine-tuned on each device for ten epochs using a batch size of 128. This personalization step is hypothesized to mitigate data heterogeneity and enhance accuracy by adapting to local data. To prevent overfitting to local data and preserve the model's generalizability – derived from training on a diverse range of participants – the duration of fine-tuning was carefully limited.

Overall, the manuscript presents a significant contribution to the field of ECG signal processing and federated learning. The study is scientifically important and practically relevant. Incorporating the suggested experiments and comparisons would further strengthen the manuscript and provide a more comprehensive evaluation of the proposed approach.

Reviewer #4

☑ *The authors proposed to use reconstruction errors and embeded vectors from trained autoencoders using federated learning on mobile phones for anomaly detection of ECG singals collected by consumer device, Polar H10 on open access dataset. They introduced also on-device fine-tuning for personalization to address the challenge posed by non-IID data. The proof-of-concept type of study seems to be promising but I think there are a few things which the authors need to address before the manuscript shall be accepted for publication.*

> Dear Reviewer,
>
> We sincerely appreciate your thoughtful comments and suggestions for improvement. We are also encouraged to see that you find the proof of concept presented in this study to be promising. We have addressed all of your concerns and hope that the revisions meet your criteria for the manuscript to be accepted for publication. Should you have any remaining concerns, we would be happy to address them as well.

☑ *1) The authors mentioned that "58.84% of the false-positive signals were classified as signals of medium quality". If I am not mistaken, the signal quality assessment was done via the SQI score proposed by reference 48. The authors did not mention the quantitative definition of what was considered as "medium quality". It would be interesting to see the results trained and tested on the "high quality" data excluding those "medium equality" ones.*

> You are correct that we did not explicitly define (medium) quality in the context of the proposed method, which is derived from the SQI score outlined in reference 48 (now reference 50). To address this, we have adopted the terminology of quality scores described in reference 50, i.e., replaced medium quality with “Barely acceptable”. For the definition of the classes (Excellent, Barely acceptable, Unacceptable), we referenced the publication by Zhao et al. (2018).
>
> using min-max normalization; (6) Assign them a quality score by applying the method of *Zhao et al. (2018)* and following their class definitions and terminologies: *Excellent*, *Barely acceptable*, and *Unacceptable* [50]. If annotations are present, the signal is cut
>
> Additionally, outlier analysis was performed after excluding signals classified as barely acceptable (previously termed “medium”) quality. This filtering criterion was incorporated into the original implementation as we were interested in this aspect as well (see attached code snippet). During the autoencoder optimization phase—which was conducted only once due to the time-consuming nature of the study – we did not exclude barely acceptable (medium) signals. This decision was based on the assumption that pathological signals might be mistakenly identified as low-quality, potentially introducing bias if excluded at this stage. However, post-training analysis using residuals and the embedding space allowed for the effective exclusion of these data without impacting/biasing the outcome.
>
> ```
> filter_noisy_signal = True
> if filter_noisy_signal:
>     mask_federated = ~((df_federated.quality == "medium"))
>     mask_personalized = ~((df_personalized.quality == "medium"))
>     df_federated = df_federated[mask_federated].reset_index(drop=True)
>     df_personalized = df_personalized[mask_personalized].reset_index(drop=True)
> ```
>
> As anticipated, the exclusion improved the results moderately to significant. For instance, the AUC for the **FT ResEmb** increased from 0.89 to 0.93. Similarly, the sensitivity, specificity, and balanced accuracy improved. These findings have been incorporated into the manuscript.

2.5 Evaluation

the balanced accuracy as an evaluation metric. To assess the impact of signal quality, the post-training analysis was repeated excluding signals classified as barely acceptable.

3.2.1 Generalized Approach

0.82 for the federated model and 0.89 for the fine-tuned model (cf. Fig 4). When removing samples with barely acceptable signal quality or less, the AUC of the best approach, i.e. FT ResEmb, increased to 0.91. At a threshold of 0.58, the fine-tuned

Table 3. Anomaly detection metrics.

Signals	Method	Threshold	Sensitivity	Specificity	Balanced Accuracy
All	Fed Res	0.32	0.7654	0.7405	0.7529
All	Fed ResEmb	0.2603	0.7873	0.7246	0.7559
All	FT Res	0.1045	0.9506	0.6813	0.816
All	FT ResEmb	0.5838	0.866	0.8028	0.8344
Excellent	FT Res	0.0542	0.9545	0.7391	0.8468
Excellent	FT ResEmb	0.4763	0.8963	0.851	0.8737
All	Cen Res	0.1266	0.8933	0.695	0.7942
All	Cen ResEmb	0.4404	0.8181	0.7922	0.8052

We have not included the following information in the manuscript to maintain focus. However, in the interest of this response, we would like to share the figures for the use of only excellent signals broken down by individuals. As expected, the numbers show an improvement in performance:

```
Device 1     Subject p00107  Threshold 0.5381      Sensitivity 0.7143     Specificity 0.9209     Balanced Accuracy 0.8176
Device 2     Subject p05484  Threshold 1.04  Sensitivity 0.96       Specificity 0.9687     Balanced Accuracy 0.9643
Device 3     Subject p06998  Threshold 0.5985      Sensitivity 0.9394     Specificity 0.9424     Balanced Accuracy 0.9409
Device 4     Subject p03984  Threshold 1.2119      Sensitivity 0.983      Specificity 0.9694     Balanced Accuracy 0.9762
Device 5     Subject p03111  Threshold 0.0357      Sensitivity 0.6146     Specificity 0.6876     Balanced Accuracy 0.6511
Device 6     Subject p04040  Threshold 0.2585      Sensitivity 0.8822     Specificity 0.9255     Balanced Accuracy 0.9038
Device 7     Subject p03013  Threshold 1.7214      Sensitivity 0.9293     Specificity 0.9267     Balanced Accuracy 0.928
Device 8     Subject p06607  Threshold 0.265       Sensitivity 0.8276     Specificity 0.8269     Balanced Accuracy 0.8272
Device 9     Subject p04219  Threshold 0.3077      Sensitivity 0.8854     Specificity 0.8681     Balanced Accuracy 0.8768
Device 10    Subject p08750  Threshold 0.627       Sensitivity 0.875      Specificity 0.9819     Balanced Accuracy 0.9285
Device 11    Subject p05665  Threshold 0.9977      Sensitivity 0.6667     Specificity 0.9183     Balanced Accuracy 0.7925
Device 12    Subject p09225  Threshold 1.302       Sensitivity 0.9868     Specificity 0.9815     Balanced Accuracy 0.9841
Device 13    Subject p08030  Threshold 1.4848      Sensitivity 0.9149     Specificity 0.9529     Balanced Accuracy 0.9339
Device 14    Subject p09886  Threshold 1.6274      Sensitivity 0.7838     Specificity 0.9639     Balanced Accuracy 0.8738
Device 15    Subject p01851  Threshold 1.2245      Sensitivity 0.8666     Specificity 0.8398     Balanced Accuracy 0.8532
Device 16    Subject p01123  Threshold 0.0107      Sensitivity 0.6538     Specificity 0.758      Balanced Accuracy 0.7059
Device 17    Subject p03043  Threshold 0.2583      Sensitivity 0.7778     Specificity 0.7543     Balanced Accuracy 0.766
Device 18    Subject p03369  Threshold 0.4583      Sensitivity 0.8703     Specificity 0.7597     Balanced Accuracy 0.815
Device 19    Subject p06829  Threshold 0.3764      Sensitivity 0.9123     Specificity 0.8904     Balanced Accuracy 0.9014
Device 20    Subject p10969  Threshold 1.1074      Sensitivity 0.9839     Specificity 0.9848     Balanced Accuracy 0.9844
```

☑ *2) From the ROC curves shown in Figure 7, the curve for FT ResEmb seemed very likely to be significantly better than the others, but the authors did not performed any statistical tests to verify it. It will be better if the authors can compute confidence intervals to the ROC curves and perform DeLong tests on the results.*

Thank you for this valuable suggestion, which has enhanced the significance and rigor of our statistical analysis. In response, we have added confidence intervals to the ROC curves and performed DeLong tests on the results. These updates have been incorporated into the manuscript and corresponding analysis scripts. On a side note, it should be noted that the p-values appeared to be very small, which may be due to the large sample size (>3.5 million).

2.5 Evaluation

The DeLong test [52] was employed to determine whether the AUCs differ significantly, testing the alternative hypothesis that the true difference in AUC is not zero. In this context, the 95 % confidence interval for each AUC was computed. The

3.2.1 Generalized Approach

The corresponding AUC are 0.816 (95 % CI: [0.8148, 0.8176]) for the federated model, 0.872 (95 % CI: [0.8708, 0.8735]) for the centralized model, and 0.888 (95 % CI: [0.8873, 0.8892]) for the fine-tuned model (cf. Fig 4). The hypothesis that there is no statistical difference between the AUCs was rejected at a significance level of $p = 0.01$.

☑ *3) For the limitation of the study, the authors did not mention that all training and testing were restricted to iphone devices. Android phones have a dominant market share globally. It can be a serious limitation for applications.*

We agree that expanding the application to include Android devices is essential for broader adoption, especially when transitioning into a patient-based study environment or moving beyond research purposes. Android phones indeed hold a dominant global market share, and not covering this platform is a limitation for real-world applicability.

However, given the prototype nature of this research, we opted to focus on a single operating system for the initial implementation to streamline development and testing. Attempting to accommodate all possible commercial devices within the initial implementation is usually infeasible unless a unified interface allows for a one-implementation-fits-all strategy. While cross-platform tools like Flutter or ReactNative exist, these do not currently support federated learning. Therefore, we decided to support only one operating system for this feasibility study. In addition, the iOS ecosystem is more homogeneous compared to the devices that run on Android. Therefore, iOS is a suitable candidate for the development of prototypes that cover a large number of devices of different generations and capacities with a single implementation. Since Flower (the used FL library) has libraries for both iOS and Android, it is theoretically possible to use a mixed-device setup. We are confident that the additional functional requirements can be adapted to the Android OS as well, and we have included this as a future research endeavor in the manuscript. To address this valid concern, we have explicitly outlined this restriction in the limitations section.

2.3 Mobile Application

Because iOS devices maintain a relatively homogeneous environment across different device generations and hardware capacities, this platform serves as a suitable basis for the prototype implementation. Since Flower also provides an Android implementation, extending the approach to a mixed-device setup remains a feasible direction for future work.

4.4 Limitations

This study was conducted using twenty mobile devices of varying generations, all operating on the iOS platform. While this provided a cross-device environment, it inherently limits the generalizability of our findings, especially considering Android's dominant global market share. Consequently, the exclusive focus on iPhone devices restricts the applicability of *Cardioflow* to a broader range of users and operating systems. Additionally, the relatively small scale of testing poses another limitation. In real-world applications, systems typically involve a much larger number of devices, which can introduce complexities such as non-IID data and potentially reduced accuracy.

☑ *4) The results on p03111 were overall worse than the others. It would be interesting to look into it and discuss what caused this difference.*

Thank you for bringing this detail to our attention. We conducted the following investigation regarding subject p03111:

1. Training Error Analysis: The mean absolute error was low and consistent across all segments, indicating no significant issues during autoencoder training.
2. Class Distribution Examination: The class distribution was as expected, with the “Normal” class being dominant.
3. Comparison with Centralized Training Results: We compared the federated learning results with those obtained from centralized training using the same data. Notably, the centralized training results for this subject were even worse.
4. Segment-Specific Error Investigation: We analyzed the error induced per segment, operating under the assumption that only a few segments might be causing the issue. Indeed, segments 34 and 49 exhibited particularly high error rates, although elevated errors were observed across all segments.

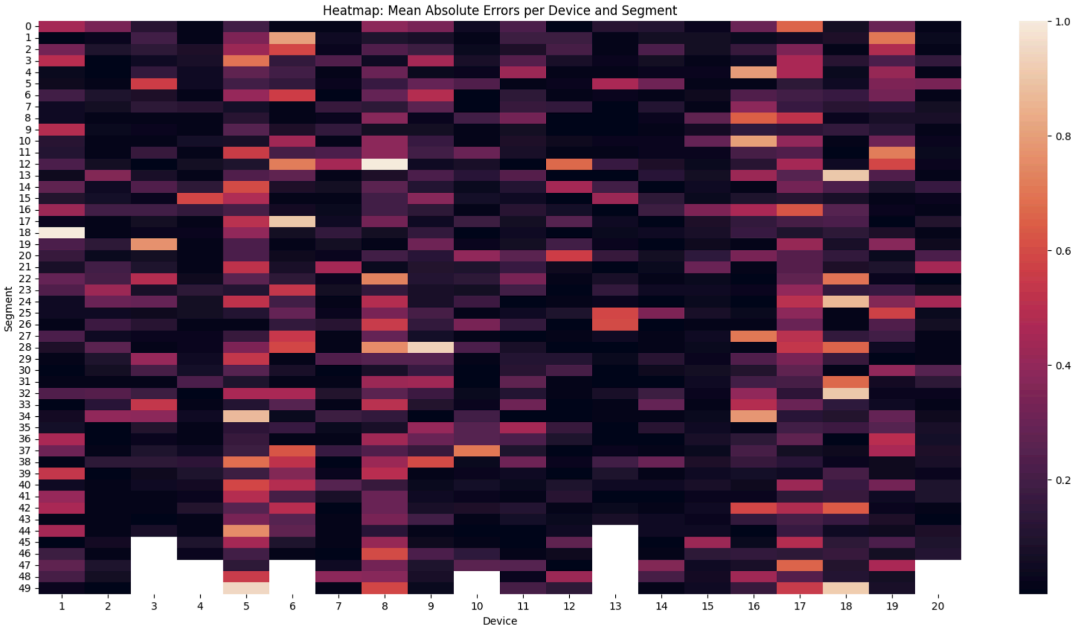

5. Detailed Signal Inspection: We closely examined the signals from Device 5, focusing on segments 34 and 49. In segment 34, we observed unusual signal behaviour, which indicates a problem in the dynamic range, which likely contributed to the poor performance for this segment. For reference, we plotted a segment from another device with better performance for comparison purposes.

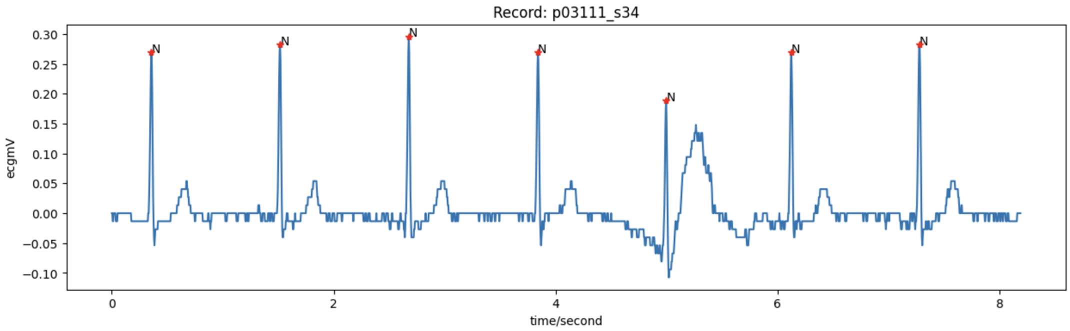

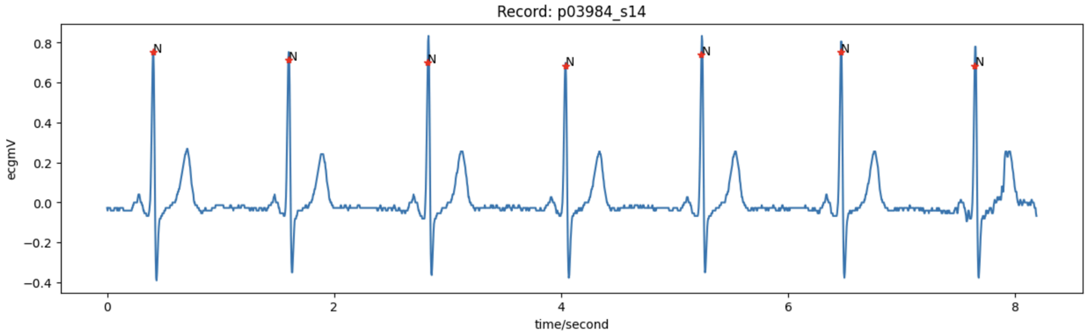

In segment 49, a similar pattern was observed, though with less intensity.

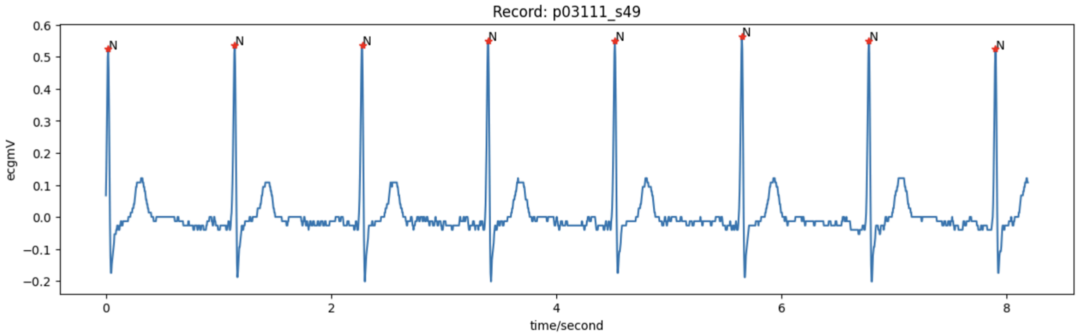

Upon further review of the Icentia11k dataset, we noticed that this pattern of “low dynamic range” appears intermittently. The excessive occurrence of this pattern in the data from p03111 may be causing the model to classify these signals as anomalous, even though they are not pathological. This non-smoothness in the signal slope seems to adversely affect the model’s performance for this subject.

We propose to explicitly mention the low performance of p03111 in the Results section and hypothesize that the root cause is related to the non-smoothness of the signal slope. We remain open to conducting further investigations to better understand and address this issue.

was below 0.8 in 2 cases. Subject *p03111* demonstrated notably poor performance, particularly in segments 34 and 49. Upon closer examination and visualization, the signals in these segments exhibited non-smooth slopes and a low dynamic range.

Reviewer #5

☑ *The authors conducted a comprehensive study on Federated Learning (FL) for ECG signal anomaly detection. They developed an iOS app to collect ECG data, train a model locally, and share model weights with a central server for global updates. The model was evaluated on unseen data from the same 20 users who participated in training, achieving a sensitivity of 0.88 and a specificity of 0.84. Several aspects require clarification:*

> Dear Reviewer,
>
> We sincerely thank you for your thoughtful comments. We believe that addressing your feedback has significantly enhanced the clarity and understanding of our approach and study. We remain committed to addressing any further concerns or clarifying any remaining questions you may have.

☑ *- How was the decision made regarding the latent vector size in the autoencoder?*

> The decision regarding the latent vector size (LVS) of 12 was informed by two key factors:
>
> Related research on employing autoencoders for single-lead ECGs frequently use latent vector sizes ranging between 8 and 24.
>
> Previous research conducted by the authors on embedding ECG data using a variational autoencoder demonstrated that an LVS of 12 provided the most optimal results.
>
> Based on these considerations, we selected an LVS of 12 for this study. We have referenced the relevant article and elaborated on this design decision in the manuscript to provide clarity.
>
> due to the limitations of the mobile machine learning framework CoreML[1]. The dimension of the latent vector space was set to $k = 12$, based on the performance demonstrated in prior research by *Kapsecker et al. (2024)* [46]. Their study highlighted the effectiveness of a 12-dimensional latent space in embedding single-lead heartbeats using the autoencoder paradigm.

☑ *- Given the sigmoid output activation, input signals must be normalized. Please elaborate on the normalization method used.*

> Thank you for drawing our attention to this detail. Indeed, the signals were normalized to the [0, 1] range using the traditional min-max normalization method, calculated as (x - min) / (max - min). We have added this explanation to the description of the methodology in the manuscript.
>
> signal to a length of 500 by linear interpolation; (5) Map the values into the $[0, 1]$ range using min-max normalization; (6) And assign a quality score to them using the method

☑ *- How would the model's performance evolve with a large number of users? Would it be continuously updated, and how would this impact the accuracy for early users?*

> The impact of an evolving number of users on the accuracy for early users is an interesting aspect that was not considered within the scope of this research. Generally, there is a hypothesis that a larger and more diverse user base contributes to a more robust model due to the increased variety of data seen during training. However, the specific effects on early users and overall accuracy remain an open question – we have acknowledged this as a

limitation of our approach.

We have added an examination of the impact of evolving client counts, both on the accuracy for early users and on general model performance, as a future research direction in the manuscript.

4.4 Limitations

limitation. In real-world applications, systems typically include a much larger and over the time evolving number of devices, which can lead to complexities such as non-IID data and potentially lower accuracy. However, this larger scale can also contribute to the development of more robust and generalizable models – an aspect not addressed in this study.

4.5 Future Work

Several limitations identified in this study provide a foundation for future efforts: (1) extending support to additional operating systems; (2) enabling federated learning routines to run as background processes, thereby enhancing usability without disrupting user activities; (3) implementing participation constraints based on device capabilities, operational state, and connectivity, as well as exploring client clustering techniques to optimize performance and resource utilization; and (4) repeating the experiment by iteratively sampling different subsets of the Icentia11k dataset and simulate an evolving number of clients over time to demonstrate robustness and generalizability, or conducting studies using a larger set of devices.

☑ *- Is there a risk of the model learning to reconstruct anomalies if it encounters a sufficient number of anomalies in the training dataset?*

Theoretically, the model could learn to reconstruct anomalies if the training dataset contains a significant proportion of anomalous data. For instance, if the application is deployed to only a small number of users who predominantly exhibit the same pathology in their heartbeats, determining anomalies using reconstruction loss would likely fail. In such cases, even anomaly detection based on the distance to the center of mass in the embedding space could be compromised. Only inter-subject changes would then manifest as deviations in embedding points along the dimensions of the embedding space.

We primarily assume that most ECGs used in the optimization routine are not pathological. Although the prevalence of cardiovascular disease is still high (according to the WHO), it can be assumed that the majority of signals are normal and “free of cardiovascular problems”. However, if a single individual contributes a large number of pathological signals, excessive fine-tuning could distort the model by overshadowing the diverse distribution learned through federated training, which includes a wide variety of ECGs from numerous subjects. Therefore, the fine-tuning process is carefully limited to a few rounds. We have revised the manuscript to clarify the rationale for limiting the fine-tuning steps and have addressed this in our response to the research question on inter-subject variations.

2.4 Technical Setting

To facilitate personalization, the base model was independently fine-tuned on each device for ten epochs using a batch size of 128. This personalization step is hypothesized to mitigate data heterogeneity and enhance accuracy by adapting to local data. To prevent overfitting to local data and preserve the model's generalizability – derived from training on a diverse range of participants – the duration of fine-tuning was carefully limited.

4.3 Inter-subject Variations

For **RQ3**, on-device fine-tuning facilitates personalization and mitigates inter-subject variation, addressing the challenge of non-IID data. However, future research needs to explore strategies to ensure that fine-tuning does not fundamentally alter the model. The valuable information learned from different subjects could disappear and affect the generalizability and overall performance of the model.

- [x] *- Could you clarify the process for determining individual anomaly thresholds?*

The individual thresholds for anomalies were calculated using ROC curves in the same way as the generalized threshold. In the individualized approach, only the individual's own data (residuals + embedding space) was used, whereas in the generalized approach, all individuals were used to calculate an overall threshold. In the Appendix, we have included the ROC curves applied to the individual data that were the source for determining the respective individual thresholds for anomalies. We hope that adding the following phrase improves the understanding in this regards:

The optimal threshold was determined using the distance-based method for each individual based on their respective ROC curves (see S3 Fig), as well as for the entire population.

- [x] *- How does the algorithm handle faster heart rates, such as those during exercise, given that 1-second signals are extracted?*

Currently, there is no specific mechanism to handle faster heart rates, such as those during exercise, where more than one R-peak might occur within a 1-second signal. Previous experiments have shown that arrhythmias like tachycardia and bradycardia tend to form distinct clusters within the embedding space when using the approach of extracting 1-second ECG signals. Faster heart rates increase the complexity that the machine learning model needs to learn. However, the general procedure of aligning R-peaks to the center of the 1-second signal provides a significant advantage by reducing the model's burden of handling variable R-peak positions within the signal.

This alignment strategy should enable the current model to capture deviations caused by faster heart rates. Nevertheless, further investigation is needed to fully address this issue. One potential approach could involve employing context-specific models tailored to different conditions, such as resting or exercising, as ECGs with faster heartbeats are likely to diverge significantly from those recorded in a resting state.

We have added this point as a direction for future work in the manuscript.

> Movement detection is often linked to exercise, which typically results in faster heartbeats and can alter the morphology of one-second ECG segments. This complexity requires the model to effectively capture these variations. By incorporating physical activity data from sources such as smartwatches and vital information like resting heart rate, it becomes possible to either exclude exercise-induced data or develop context-specific models. This enables precision approaches tailored to specific situations, e.g., resting versus exercising. Also, forming client clusters based on characteristics such as average heart rate and demographics can enhance these precision approaches by aligning and comparing participants more closely in their ECG data distributions.

- [x] *- Why were annotations transferred to devices, while they should be needed only for evaluation of the results?*

For the scope of this study, there was no specific necessity to transfer annotations to the device beyond enabling visual examinations (as shown in Figure X - Client (1)) and on-device verifications. Initially, we considered the possibility of leveraging semi-supervised approaches to identify pathologies, such as by clustering ECGs and labeling a few representative samples within each cluster to infer the class of the entire cluster. This idea influenced the decision to retain the functionality of injecting or manually annotating data on the device.

While this functionality was not essential for the current study, we chose not to remove it, as it could prove useful in future explorations. We have ensured that the manuscript clarifies the role of annotated data on the device to avoid any potential confusion.

> (3rd generation), 1 x iPad Air (4th generation). The CSV files containing the electrocardiogram signals and annotations were transferred to the devices and stored in the application's document folder. The annotations were required solely for demonstration purposes, such as the visualization of clustering behavior illustrated in Fig 3 Client (1). The uploaded ECGs were preprocessed and segmented according to

- [x] *- Using the first segments for testing might introduce bias due to learning effects or device adjustments.*

The 50 segments used for testing were randomly sampled from a larger set of patient segments, as specified by the Icentia11k dataset: “We segment each patient record into segments of [...] ~70 minutes. [...] From this, we randomly select 50 of the segments and their respective labels from the list of segments. The goal here is to reduce the size of the dataset while maintaining a fair representation of each patient.” As a result, the first segment used in testing is unlikely to correspond to the chronological first segment.

To clarify this point, we have added the above explanation to the manuscript in the section where we discuss using the first segment as the test set.

> During each round, five optimization epochs took place on the device. The test set was set to the first segment per subject, while the remaining segments were used for training. As the dataset creators randomly selected fifty segments from a larger pool, the first segment is unlikely to represent the participants' initial recording. This approach ensures that the test data are free from bias due to initial learning effects and device adjustments. Loss was calculated using mean squared error and recorded after

☑ *- Please clarify the meaning of "unknown beats" and whether they were excluded from the study. Could normal and anomalous beats be inferred from rhythm annotations?*

Yes, this requires clarification and is in its current form not well explained. The beat labels include Normal, PAC (Premature Atrial Contraction), PVC (Premature Ventricular Contraction), Undefined, and “+”, where the “+” symbol indicates a rhythm annotation at a specific location. However, for the purposes of our analysis, rhythm labels were excluded because the focus was on individual heartbeat segments. Although arrhythmias can alter the morphology of single heartbeats, their reliable detection typically requires analyzing consecutive heartbeats. Therefore, from a medical perspective, annotating arrhythmias based solely on single beats is not meaningful. While an arrhythmia might coincidentally occur simultaneously with a heartbeat, the independent validity of beat annotations ensures that this should not impact the analysis. We have reworded the relevant part to mitigate the wording “unknown beats”:

> columns. The annotation ”+” indicating a rhythm label was ignored for later analysis and treated as no annotation. Although arrhythmia can alter the morphology of single-lead ECG heartbeats, accurate identification typically requires analyzing consecutive heartbeats. Therefore, using arrhythmia annotations based solely on single beats may result in medically unreliable samples. *Normal*, *PAC* and *PVC* were the only

☑ *- Could you define the embedding vector and embedding space used in the autoencoder?*

We have thoroughly revised the autoencoder section to provide a more comprehensive description, explicitly incorporating the terms “embedding space” and “embedding vectors”.

> The architecture of an autoencoder consists of two stacked neural networks, the encoder E_ϕ and the decoder D_θ, parameterized by the weights ϕ and θ, respectively. The encoder maps data from a high-dimensional space $V \subset \mathbb{R}^n$ into an embedding space $L \subset \mathbb{R}^k$ of low dimension, where $k \ll n$, formally expressed as $E_\phi : V \to L$. The low-dimensional representations $l \in L$ are referred to as the embedding vectors. In a standard setting, the decoder $D_\theta : L \to \mathbb{R}^n$ attempts to reconstruct the original input from the embedding vector. Accurate reconstruction is achieved if the k latent dimensions effectively encode the predominant signal information. Thereby, the autoencoder is optimized by adjusting the weights ϕ and θ to minimize the reconstruction error through an appropriate loss function.

☑ *- It would be nice to analyze the results on how increasing the number of users can affect the results. The model can be saved and used for another set of 20 users, right?*

You are correct that the model can be saved and subsequently used for another set of 20 users. However, as noted in previous responses, our current study faced practical limitations that constrained our ability to conduct such extensive evaluations:

1. Manual Data Handling: The process of transferring and loading subject data from the Icentia11k dataset onto each device required manual intervention. Similarly, establishing connections to the federated learning server and executing training routines involved manual steps. Automating these processes was beyond the scope of this prototype implementation, making experiments with varying client numbers highly time-consuming and resource-intensive.

2. Device Availability: The limited availability of devices further constrained our ability to

conduct multiple training runs with varying client configurations. Coordinating additional sessions to explore different user counts was not feasible within the project's timeframe.

Despite these constraints, we took alternative steps to ensure the robustness of our findings:

Confidence Intervals: With the initial sample of 20 devices, we calculated confidence intervals for the AUCs for the different approaches using DeLong Test, demonstrating the statistical significance and superiority of the fine-tuning approach.

Centralized Model Training: We also trained a centralized model using the 20 samples. This comparison highlighted any potential discrepancies between federated and centralized training paradigms, reinforcing the validity of our federated approach.

We recognize the importance of assessing the impact of increasing numbers of users in the federated learning process and have included this as a key direction for future work. We have indicated the changes we have made in an earlier response when you commented: How would the performance of the model evolve with a large number of users?

Thank you once again for your constructive feedback. We are committed to further improving the manuscript and look forward to addressing any additional comments you may have.

Reviewer #6

☑ *Overall Evaluation:*

The authors present an interesting proof-of-concept study exploring the use of federated learning with autoencoder models for detecting cardiovascular anomalies from single-lead ECG data in a mobile environment. The study shows promise for potential applications in mobile health, especially for consumer health, where it could help detect ECG anomalies via mobile-phone-based applications. However, there are several key areas where the study's generalizability, methodology, and validation need further clarification or improvement. These issues are outlined below and should be addressed to strengthen the manuscript.

> Dear Reviewer,
>
> Thank you for your thoughtful feedback, which has significantly strengthened our manuscript. We acknowledge your assessment that the model's generalizability is limited in its current form, primarily due to the availability of devices and the prototype nature of the study. However, we believe this work represents an important initial step toward future patient-based research. We have revised the manuscript accordingly to realistically reflect these limitations and hope that our revisions meet your expectations for publication.

☑ *1. Generalizability of the Model:*

The dataset used to train the model appears to come from a population with an average age of 62 years, according to the source referenced (https://physionet.org/content/icentia11k-continuous-ecg/1.0/). There is no discussion of the diversity of the dataset, particularly in terms of race and sex, which is crucial for understanding the model's performance across various demographic groups.
As the model's performance is highly dependent on the training data, the lack of demographic diversity may limit its generalizability. The authors should clarify this in the manuscript and, if applicable, acknowledge this as a limitation.

> We agree with the reviewer that demographic information is highly relevant when it comes to the generalizability of the approach. Therefore, we have added available information and metadata regarding the Icentia11k dataset.
>
> The dataset was primarily collected from 2017 to 2018 in Ontario, Canada, comprising 45.3% female, 42.6% male, and 12.2% participants with unspecified gender. The age distribution approximates a normal (bell-shaped) curve, with a mean age of 62.2 years and a standard deviation of 17.4 years. Although there is an average inclination towards an older population, the ages range widely from adolescents to individuals close to 100 years old. The long-term recordings consist of single-lead ECG signals collected over 3
>
> We also acknowledged this fact in the limitation section:
>
> coronary syndrome, myocardial infarction, and left ventricular hypertrophy [4]. The demographics of the data set, in particular the proportion of participants in last third of life, also represent a limitation to generalizability.

☑ *2. Inter-Subject Variability:*

The authors acknowledge significant inter-subject variability in their analysis, as indicated by distinct clusters in the embedding space based on individual subjects. This suggests that

ECG signals vary significantly across individuals, potentially impacting the model's sensitivity and accuracy when applied to populations outside the demographic represented in the training data.
The manuscript should either address how the model handles this variability or clearly state this as a limitation. Additionally, the demographic distribution of the users of the 20 mobile devices tested should be disclosed to assess the impact of these differences on the results.

We acknowledge that, in its current form, the generalizability of our model is limited. This aligns with our upcoming responses to points **3. Limited Device Testing**, **4. Lack of Annotation Clustering**, and **6. Lack of External Validation**, where we discuss inter-subject variability, the steps we have taken to address this issue, prevailing limitations, and future work to address these concerns. To streamline our response, we kindly refer you to those detailed explanations.

Regarding the demographics of the individuals, we attempted to access this information but, unfortunately, it is not available in the published data—only overall demographics are provided, as outlined in our response to **1. Generalizability of the Model**. Sampling twenty individuals from a dataset of 11,000 may not accurately reflect the overall demographics, and we acknowledge this as a limitation of our study.

The demographic composition of the dataset, particularly the overrepresentation of participants in the later stages of life, present a limitation to the generalizability of the findings. Additionally, the twenty subjects randomly sampled from the large pool of 11, 000 participants may not accurately reflect the overall demographics. However, since the dataset did not provide demographic information on an individual subject basis, only general statements can be made about the demographics of the selected samples.

☑ *3. Limited Device Testing:*

The proof-of-concept was only tested on iPhone users, which raises concerns about the generalizability of the tool to users of other devices or operating systems. This should be addressed by either expanding testing to a broader range of devices or including this limitation in the manuscript.

We agree that expanding testing to include Android devices is essential for broader applicability. Android phones hold a dominant global market share, and not covering this platform limits the tool's reach. However, given the prototype nature of this research, we opted to focus on a single operating system to streamline development and testing. We are confident that the functional requirements can be adapted to the Android OS, and we have included this as a future research direction in the manuscript. This limitation has been explicitly acknowledged in the limitations section. Please refer to our response to comment **6 Lack of external validation**, as we have combined these two aspects into one limitation. However, we provided a rational for implementing the prototype for iOS first:

2.3 Mobile Application

Because iOS devices maintain a relatively homogeneous environment across different device generations and hardware capacities, this platform serves as a suitable basis for the prototype implementation. Since Flower also provides an Android implementation, extending the approach to a mixed-device setup remains a feasible direction for future work.

☑ *4. Lack of Annotation Clustering:*

In Section 3.2, the authors note that no significant clustering was observed when the embedding space was colored by annotations (e.g., normal, PAC, PVC), indicating that inter-subject differences overshadow the variations between different types of heartbeats within individuals.
This raises concerns about the tool's ability to detect anomalies within a single subject's ECG. While the authors suggest that fine-tuning the model to individual subjects could improve accuracy, they need to demonstrate this with data, especially for subjects who do not fit within the distribution of the training data. If not, this should be listed as a limitation of the approach.

> Thank you for raising this concern, which aligns with your comments on **2. Inter-Subject Variability** and **6. Lack of External Validation**. We agree that, in its current form, the generalizability to unseen data may be limited due to insufficiently learned data distributions. Our assumption is that each participant takes part in the federated learning process, allowing the model to learn their respective data distribution. However, we acknowledge that capturing all possible ECG data distributions may not be feasible with a single model, and a more complex model might be required.
>
> Therefore, precision approaches should be considered. This includes forming client or situation clusters that exhibit similar ECG characteristics – an area yet to be explored. These characteristics might include resting heart rate, heart rotation/position, or contextual factors such as whether the user is exercising or at rest. We have added this important point to our future work section, proposing it as an intermediary approach between training a single model over all participants and fine-tuning individual models.
>
> requires the model to effectively capture these variations. By incorporating physical activity data from sources such as smartwatches and vital information like resting heart rate, it becomes possible to either exclude exercise-induced data or develop context-specific models. This enables precision approaches tailored to specific situations, e.g., resting versus exercising. Also, forming client clusters based on characteristics such as average heart rate and demographics can enhance these precision approaches by aligning and comparing participants more closely in their ECG data distributions.
>
> Additionally, we carefully limited the number of fine-tuning epochs to ten to prevent the model from overfitting to an individual's data distribution. We have included this detail in our explanation to clarify our intention.
>
> To facilitate personalization, the base model was independently fine-tuned on each device for ten epochs using a batch size of 128. This personalization step is hypothesized to mitigate data heterogeneity and enhance accuracy by adapting to local data. To prevent overfitting to local data distribution and preserve the model's generalizability – derived from training on a diverse range of participants – the duration of fine-tuning was carefully limited.

☑ *5. High False Positive Rate:*

The authors should explicitly mention the high false positive (FP) rate observed during testing as a limitation. Additionally, strategies to mitigate this issue, or adjustments to the tool's intended goal to account for this, should be discussed.

> Indeed, this is an important topic that requires consideration. We have expanded our research, such that we also analysed the impact on the results when excluding signals of low

quality (as measured by SQI introduced by Zhao et al. (2018)) during the post-analysis. The finding is an improved performance, particularly for the specificity which is directly linked to the FP.

Table 3. Anomaly detection metrics.

Signals	Method	Threshold	Sensitivity	Specificity	Balanced Accuracy
All	Fed Res	0.32	0.7654	0.7405	0.7529
All	Fed ResEmb	0.2603	0.7873	0.7246	0.7559
All	FT Res	0.1045	0.9506	0.6813	0.816
All	FT ResEmb	0.5838	0.866	0.8028	0.8344
Excellent	FT Res	0.0542	0.9545	0.7391	0.8468
Excellent	FT ResEmb	0.4763	0.8963	0.851	0.8737
All	Cen Res	0.1266	0.8933	0.695	0.7942
All	Cen ResEmb	0.4404	0.8181	0.7922	0.8052

We believe that excluding low quality signals at the time of recording already improves the overall performance of the system. However, there are some things to consider, e.g., excluding low-quality signals could systematically exclude anomalous heartbeats due to the potential correlation of quality score and annotations - however, this was not observed in this study with a Pearson correlation coefficient of 0.0071.

quality. The correlation between the quality and the annotations was not significant with a Pearson correlation coefficient of 0.0071. All devices had at least one anomalous

Other strategies to mitigate FP include the exclusion of signals when fast movements are detected simultaneously, e.g. through additional measures such as IMUs. Pursuing a precision approach using models for different situations (e.g. resting vs. exercising) is also subject to these strategies. We have summarized these aspects in the Future Work section:

The promising results of this study encourage further research using real-time measurements with devices such as the Polar H10 and FL in a more unobtrusive fashion. In this context, automatically excluding low-quality signals is expected to enhance performance, particularly specificity due to a lower count of false positives, as demonstrated in this study when signals of barely acceptable quality and lower were omitted. However, it is important to investigate the impact of pathologies on quality measures to ensure that no bias is introduced by systematically excluding pathological ECGs from the analysis. To avoid motion-related distorted ECG readings, it is important to analyze the IMU data simultaneously to exclude segments where sudden movements are detected that can potentially distort the ECG recordings. It is expected that implementing this strategy will reduce type II errors and improve the specificity and precision of the results.

Movement detection is often linked to exercise, which typically results in faster heartbeats and can alter the morphology of one-second ECG segments. This complexity requires the model to effectively capture these variations. By incorporating physical activity data from sources such as smartwatches and vital information like resting heart rate, it becomes possible to either exclude exercise-induced data or develop context-specific models. This enables precision approaches tailored to specific situations, e.g., resting versus exercising.

☑ *6. Lack of External Validation:*

The model was developed and tested on a specific dataset, with no evidence provided for external validation. The lack of external validation is a critical concern for a model intended for real-world deployment. The authors should either provide evidence of external validation or clearly state this as a limitation of the study.

> Despite being trained on over 3.5 million ECG traces, we acknowledge that our model in its current form is probably not generalizable to all individuals and ECG variations due to the limited number of participants (n = 20) involved in the training process. A major advantage of the proposed system is its ability to grow with the number of users until a critical mass is reached, at which point the model can be considered generalizable. This should at least hold true for all participants in the federated learning model, as it should capture their individual data distributions, which are likely to resemble those of new users as the participant pool expands.
> Since the system is unsupervised, the primary limiting factor is the number of participants rather than the availability of healthcare professionals to label data. However, incorporating a few annotations in the embedding space could further improve accuracy (see Future Work). To address the concern that the model cannot yet be applied to ECGs from unseen individuals, we have elaborated on this in the Limitations section, where we discuss the generalizability constraints of our approach.
>
> This study was conducted using twenty mobile devices of varying generations, all operating on the iOS platform. While this provided a cross-device environment, it inherently limits the generalizability of our findings, especially considering Android's dominant global market share. Consequently, the exclusive focus on iPhone devices restricts the applicability of *Cardioflow* to a broader range of users and operating systems. Additionally, the relatively small scale of testing devices poses another limitation. In real-world applications, systems typically include a much larger and over the time evolving number of devices, which can lead to complexities such as non-IID data and potentially lower accuracy. However, this larger scale can also contribute to the development of more robust and generalizable models – an aspect not addressed in this study.

The study provides an innovative approach to ECG anomaly detection in a mobile setting. However, addressing the concerns related to generalizability, inter-subject variability, false positives, and external validation is crucial for the broader applicability of the tool. The authors should either address these issues or acknowledge them as limitations in the manuscript.

I look forward to the revision.

Reviewer #7

- [x] *Dear authors, thank you for your very interesting manuscript. Overall the paper reads very well. However, I would suggest some improvements, especially in terms of readability, but also to clarify the study's test status.*

> Dear Reviewer,
>
> We sincerely thank you for your valuable feedback and thoughtful comments. Your suggestions have been a great support in enhancing the quality of our manuscript. We have worked dedicated to addressing all of your comments and remain committed to resolving any additional concerns you may have. We hope this revision meets the reviewers' criteria for publication.

- [x] *Introduction*
  - The introduction is a little overwhelming to read if you want to get a quick overview of the essence of the work. I would suggest starting with a short introduction that quickly leads to the objective and research questions (both of which I would assume are at the end of the introduction), and move the literature review and descriptions of related work to another subsection.

> Following your recommendation, we have reorganized the 1 Introduction into three subsections:
> **1.1 Overview & Motivation** Introducing the topic, objectives and short outline
> **1.2 Related Work** Stating about related work based on the objectives
> **1.3 Research Questions** As the research questions are predominantly determined by the gap identified in existing research, we placed them beyond the end of the related work section

- [x] *- You define three research questions and address them in the paper. Since you added them, I would assume you give a summary per question in the discussion: Were each of these questions answered successfully? You could also organise the results more clearly based on these questions to make them easier to follow.*

> Thank you for this insightful suggestion, which indeed enhances the clarity and reading flow of the manuscript. We have structured the results and discussion sections around the research questions, using them as a guiding framework. We have therefore added the headings of the Results/Discussion sections to the research questions to facilitate reference. This ensures that the reader can consistently refer back to the research questions for context and understand how each question has been addressed throughout the manuscript.
>
> **RQ1** Feasibility: How feasible is autoencoders implementation for federated unsupervised learning, in a cross-device real-world scenario?
>
> **RQ2** Anomaly Detection: What is the performance of autoencoders in the detection of electrocardiogram anomalies as part of a cross-device federated unsupervised environment?
>
> **RQ3** Inter-subject variations: Can on-device fine-tuning be sufficient for personalization and detecting baseline changes, thus addressing the challenge of non-IID data?
>
> We have also repeated the research questions explicitly in the discussion section and gave concise answers:

4.1 Feasibility

The answer to **RQ1** is that federated unsupervised learning in smartphones emulating a real-world environment is computationally feasible. However, the transition from a prototype to a production-ready implementation, particularly for use in clinical trials, needs to be addressed in future work.

4.2 Anomaly Detection

The answer to **RQ2** is therefore that the accuracy of the presented approach for the given pathologies can compete with related work, but with the advantage that it is unsupervised and preserves privacy.

4.3 Inter-subject Variations

For **RQ3**, on-device fine-tuning facilitates personalization and mitigates inter-subject variation, addressing the challenge of non-IID data. However, future research needs to explore strategies to ensure that fine-tuning does not fundamentally alter the model. The valuable information learned from different subjects could disappear and affect the generalizability and overall performance of the model.

☑ *- Table 1 somehow looks like a skewed picture. Is it just the font used?*

The appearance of Table 1 as a skewed picture was due to the resizing of the font to ensure the table fits into textwidth. To fix this, we have adjusted the font and placement of the table (shifted it to the left). The final placement and size may still change in the typesetting phase – if accepted.

Table 1. Studies using FL for the detection of ECG anomalies.

Study	Datasets*	Ta
Goto et al. (2022) [14]	Self-collected from four hospitals	Ir
Sakib et al. (2021) [15]	MIT-BIH Supraventricular Arrhythmia [25, 26], MIT-BIH Arrhythmia [26,27], INCART 12-lead Arrhythmia [26], Sudden	Ultra

☑ *Methods*

- All figures: The figures have a title, but usually no description. Normally, an figure should be self-explanatory through its title and labelling.

We have revised all the figures to include titles and comprehensive labels, ensuring they are self-explanatory and provide sufficient context without requiring additional explanation.

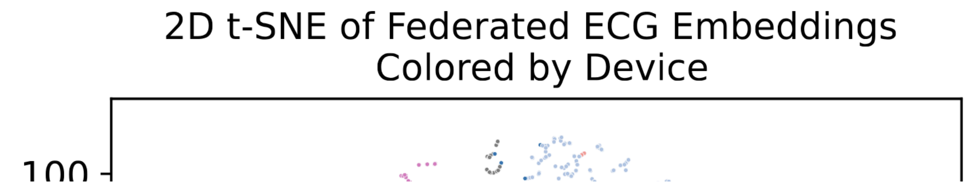

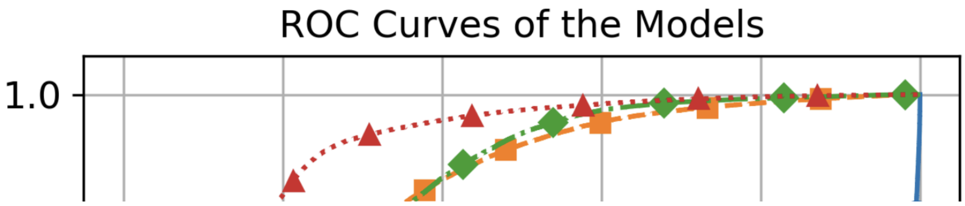

☑ *- P6, L153: Something with the wordings seems to be wrong here.*

> Fixed.
>
> Likewise, the embedding space can serve as a basis for the detection of anomalies. It is expected that most ECGs fall into ~~in~~ a nearby region due to the similarity and

☑ *- P6, L159: Maybe its better to reference the git repository here instead to the current location*

> We agree that a reference to the Git repository should be included at this specific location in the manuscript. Since the repository also contains other components relevant to Section 2, "Materials and Methods" – such as code for data (down)loading and evaluation – we initially provided the reference at the beginning of that section. However, recognizing the importance of guiding readers to the exact location within the repository where the mobile application implementation is found, we have now added a specific reference here as well.
>
> A prototype of a mobile application called *Cardioflow*[2] was developed to consider a realistic cross-device FL setting. The target platform was iOS, mainly using the Swift implementation of the Flower framework [46, 47]. In a first step, the functional requirements (FR) were defined:
>
> 1. **FR1** The application must be able to record ECG data directly from the Polar
>
> [2] `https://github.com/CardioKit/cardioflow/tree/main/src/application`. Retrieved on: 17 November 2024.

☑ *- Figure 3: You are showing a lot of screenshots. Please add some descriptions of each to the caption.*

> Fixed.
>
> **Fig 3.** The main views of the Cardioflow application, including the home screen (Client 1) displaying general statistics and analysis visualizations, the ECG recording and uploading interface (Client 2), the heartbeat reconstruction metrics (Client 3) showing model performance, and the settings panel (Client 20) for managing data processing and federated learning routines.

☑ *- Section 2.3: I would strongly suggest to use a consistent wording for watch and smartphone. I think all FR are targeted at "your" phone-App, but maybe you also have FR for the watch itself or the watch-App on the phone?*

> You are correct that all functional requirements (FR) are targeted at the phone application. We have revised the text to ensure consistent wording and to clearly specify that the FR pertains to the phone's application. In particular, we have removed any reference to the watch as it is not relevant to the study. E.g.:
>
> A prototype of a smartphone application called *Cardioflow*[2] was developed to consider a

☑ *- Section 2.3: Since this is not a development white paper, it is not really of interest that you successfully implemented the FR you defined beforehand. I would suggest to just write the features you implemented.*

> We agree that detailing both the functional requirements (FR) and their resulting implementation can be redundant. However, we believe that presenting the FR initially

provides a clear framework that enhances the reader's understanding of our study's objectives. To address your suggestion, we have revised Section 2.3 to focus exclusively on the implemented features, ensuring that only the most relevant functional requirements are included. This mainly affected FR1.

1. **FR1** The application must be able to ~~record ECG data directly from the Polar H10 device, collect existing ECG data from the device's health application, and upload~~ receive an ECG signal – in a common format such as comma separated values – including the option to provide annotations for visualization purposes. The collected data must be stored in an application-internal memory.

☑ *- Section 2.4: Is not the whole paper the study? This section might better be titled with "technical setting"*

Done.

2.4 Technical Setting

Cardioflow was deployed on twenty mobile devices running iOS version 17.5.1: 8 ×

☑ *- Figure 4 and 5 seem not to be so important. Figure 4 is similar to Figure 3 and could probably be removed or moved to the online supplement*

Done. We merged figure 4 and 5 into one and moved it to the supplemental material.

S7 Fig. Study setting. Depicts the study setting, including the devices (excluding tablets) and the console input and output of the server.

☑ *- L207ff: Did you use the FL customization functions for this study? Is this really useful instead of a common-server-provided configuration?*

Yes, we used the default server-provided configuration, which was most suitable for our study. Although the fine-tuning phase after federated learning allows for independent setting of training parameters, we applied uniform parameters (epochs = 10 and batch size = 128) across all devices. We have revised the manuscript to clarify that no customizations were made during the federated learning process and that fine-tuning was consistently performed with the same parameters on all devices.

Machine learning routines can be triggered both within a device and in a federated setting. Participating as a client node in an FL environment using the *Flower* framework requires the input of the IP address and port of the orchestrating server [47]. For subsequent on-device model fine-tuning, two arguments can be adjusted to parameterize the local model optimization: the number of epochs and the batch size. Additionally, a static threshold can be set to compute the outliers from the embedding space and the reconstruction error, as outlined in subsection 2.2. Note that these parameters are typically not actively set by the client device but are instead computed, predefined as static properties, or provided by a central server. However, to explore different parameterizations and their associated behaviors within this study, we have included the ability to adjust these parameters on the fly.

☑ *- In the 'Description of the app' section, some details of the actual calculation are described that could/should rather be presented in the 'Study' or 'Evaluation' section. For example, the fact that the snippets are cut by 1 second is described under 'Settings "View"', which is not directly understandable, since it is also relevant for the analysis.*

We agree that this fact would also fit in the “Study” section. However, when describing the mobile application, we feel that describing the full functionality is beneficial to the narrative and guiding the reader through the document. To address your comment, we have included the specific purpose of the R-Peak as a prospect in the “Mobile Application” section and have referenced back to these components within the “Study” section. We have also renamed the section “Study” to the more specific term “Technical Setting”, which is narrower, as according to another reviewer's comment, the entire work is the study.

2.3 Mobile Application

detection; (3) Cut the signal into one-second segments around the R-peak to extract single heartbeats, which are the subject of this study; (4) Upsample the heartbeat

2.4 Technical Setting

The uploaded ECGs were preprocessed and segmented according to the described functionality in 2.3 by clicking on *Start Segment ECG* in the settings tab – see Fig 5 for the experiment setting.

☑ *Results*

- In the methods you write that the data comes from Physionet. In L276ff you say that they were extracted from the 20 devices. Could you clarify this? If they come from the devices: on which subjects were they measured? Would an informed consent be necessary?

Thank you for pointing out this inconsistency and lack of clarity in our writing. To clarify, no data were obtained directly from the devices, such as through a study involving participants, which would indeed require informed consent and ethical approval. The data used in this work were solely sourced from Physionet. We have revised the paragraph to ensure it is concise and accurately reflects this.

After deploying the long-term recordings of twenty subjects to the mobile devices, a total of 3,717,142 ECG heartbeat segments were extracted on the devices from these recordings. Of these, 59,460 samples represented anomalous ECGs, either PAC

☑ *- L293ff: The restriction should perhaps be moved to the discussion.*

We agree that this point should also be included in the limitations section. We included it as a result within the feasibility section as it is an important result in regards to feasibility and practicality. While we have shown that federated learning on smartphones is technically and accuracy-wise feasible, to reliably answer the initial research question, we must also mention this aspect in the result section. However, we also emphasized and extended on this point in our limitation section.

The iOS ecosystem prohibits calling a function remotely when the application is closed, which makes unobtrusive and randomly scheduled FL as a background task currently infeasible. This limitation requires the user to be online and actively participating as a client node.

☑ *- Section 3.3: You take up the method description here. But you are actually describing the evaluation scenario again, which should somehow be defined in the Evaluation section. I would suggest defining a specific table in the Method Evaluation section that describes the actual models you used for the whole evaluation (i.e. as a specification for Table 2). The*

whole methodology should be understandable without reading the results. The results could be written in a more condensed way.

Thank you for your suggestion to improve the model description and reduce redundancy by avoiding repeating parts of the evaluation method. We have taken up your suggestion and introduced a table explaining the models, their abbreviations, and their purpose.

Table 2. Autoencoder model variants for the evaluation of the ECG anomaly detection.

**Identifier**	**Setting**	**Description**
Fed Res	Federated	Base autoencoder trained on data distributed to 20 devices. Anomalies identified using reconstruction residuals.
Fed ResEmb	Federated	Base autoencoder trained on data distributed to 20 devices. Anomalies identified using residuals and embedding vectors.
FT Res	Fine-tuned	Fine-tuned the base model locally on each device to adapt to individual data distributions. Anomalies inferred using residuals.
FT ResEmb	Fine-tuned	Fine-tuned the base model locally on each device. Anomalies inferred using residuals and embedding vectors.
Cen Res	Centralized	Autoencoder trained on centrally stored data. Anomalies identified using residuals.
Cen ResEmb	Centralized	Autoencoder trained on centrally stored data. Anomalies identified using residuals and embedding vectors.
