# Supplementary material for: Cross-device federated unsupervised learning for the detection of anomalies in single-lead electrocardiogram signals
Source: PLOS Digit Health. 2025 Apr 7;4(4):e0000793. doi: 10.1371/journal.pdig.0000793 (PMC11975069; doi:10.1371/journal.pdig.0000793)
Supplement: Attachment — Submitted filename: Response_to_Reviewers_auresp_2.pdf [file pdig.0000793.s011.pdf]

Cross-device federated unsupervised learning for the detection of anomalies in single-lead electrocardiogram signals

Response to Reviewer Comments II

PDIG-D-24-00344

We thank the reviewers for their detailed comments, which have greatly helped to improve the manuscript. Below, we provide a point-by-point response to the open comments.

Reviewer #1

- [x] *The authors have implemented my suggestions for the manuscript and the supporting information in a convincing way. I recommend to accept the manuscript after a minor revision (referring to line numbers in the "clean" version of the revised manuscript):*

> Dear Reviewer,
> Thank you for acknowledging our revisions and for your additional suggestions to improve our manuscript. We have addressed them as follows:

- [x] *l 217: I would suggest framing this slightly differently to distinguish what is necessary in the present prototype from how it would be used in later routine, along the lines of "in the present implementation, ECG data is uploaded as a CSV file ..."*

> Thank you for your comment. In response, we emphasized that uploading CSV files is part of the current implementation (study), while recording via a consumer device (which has already been implemented but not yet used) is intended for a follow-up study.
>
> In the present implementation, ECG data is uploaded as a CSV file~~Users can upload a signal from a CSV file~~, which can also contain predefined annotations. Looking ahead to future work involving a patient-based study, it is intended~~also possible~~ to record an ECG using the Polar H10 device as a data source.

- [x] *l 260 should read "in Section 2.3" (or actually reference the respective subsection by title, I don't think PLOS Digital Health uses numbered sections, please double-check)*

> Thank you for bringing this to our attention. We could not find an explicit statement on how to reference (sub)sections within the manuscript. Therefore, we proceeded as we would reference content from the supplementary material (i.e. using \nameref{}), as also suggested in your comment, by saying “reference [...] by title”. For printed versions without the hyperref marker, we added the identifier “Section”. We have also adapted l 274 in this respect.
>
> space and the reconstruction error, as outlined in Section Autoencoder for Anomaly Detection. Note that these parameters are typically not actively set by the client device
>
> described functionality in Section Mobile Application by clicking on *Start Segment ECG* in the settings tab – see S2 Fig for the experiment setting.

- [x] *l 334 and possibly later locations where signal quality is mentioned: I would suggest repeating "[51]" to avoid the impression that this is some ad-hoc assessment. The classification is described as part of the Methods, but rather hidden in the text (which I think is fine).*

We agree that this wasn't sufficiently highlighted. Following your suggestion, we added the reference each time signal quality is mentioned, including at line 334:

the post-training analysis was repeated excluding signals classified as *barely acceptable* and lower [51]. The results of the subject-based analysis as well as insights into the

- [x] *l 422 and 515: I would suggest replacing "significant" with "substantial" in contexts not describing statistical significance to avoid potential confusion.*

Done.

of such an approach without substantially~~significantly~~ affecting device functionality on

dropping out due to network instability or power loss can substantially~~significantly~~

- [x] *l 503: I would suggest phrasing the limitation on iOS devices slightly differently, along the lines of "our current focus on iPhones", in order to avoid suggesting the approach is generally impossible on Android or other platforms.*

We agree that this point should be framed as a limitation of generalizability of the findings rather than a restriction on transferability to different operating systems. Accordingly, we have revised the paragraph.

This study was conducted using twenty mobile devices of varying generations, all operating on the iOS platform. While this provided a cross-device environment, it ~~inherently~~ limits the generalizability of our findings to other operating systems such as Android~~, especially considering Android's dominant global market share~~. The current ~~Consequently, the exclusive~~ focus on iPhone devices does not allow for statements on accuracy and performance~~restricts the applicability~~ of *Cardioflow* when simultaneously deployed to different~~a broader range of users and~~ operating systems. Additionally, the

- [x] *l 514: I would suggest adding a literature reference after "Goldberger lead I" for readers unfamiliar with this term.*

Thank you for bringing this to our attention. We mistakenly referred to Goldberger lead I instead of Einthoven's lead I. In single-lead ECG measurements from wearable devices, Einthoven's lead I (or a modified version of it) is typically used. However, to avoid confusion, we now use the more general term "single-lead ECG" and cite Einthoven's lead I as a specific example for measurement. In response to your comment, we have also included a reference to the original publication in which Einthoven introduced his convention.

atrial fibrillation, which might be a more important topic for investigation. Also, some pathologies may not manifest in individual~~single~~ heartbeats when only a single-lead ECG signal (e.g., Einthoven's lead I [53]) is examined. Therefore, not all potential

- [x] *l 544: I would suggest rephrasing to "with heart frequency sensors such as the Polar H10".*

Done.

The promising results of this study encourage further research using real-time measurements with heart frequency sensors~~devices~~ such as the Polar H10 and FL in a

Reviewer #4

The finding of non-smoothness of signals might explain the lower performance of the model on subject p03111 is interesting. For non-expert readers about ECGs like me, it might be good to show a few typical cases of how pathologically relevant abnormal ECG signals should look like in the supplementary material. Other than that, I don't have more concerns about the updated manuscript.

Dear Reviewer,

Thank you for reviewing our revision and suggesting additional ECG samples as supplemental material. We agree that more examples illustrating each pathology help readers understand how these conditions shape the ECG waveform and how they differ from one another. In response, we have added 12 randomly sampled ECG snippets (four for each label) to the supplemental material and referenced them in the main text.

normal, premature atrial contraction (PAC), premature ventricular contraction (PVC), or undefined, i.e., unclassifiable beat (cf. Fig 1 and S1 Fig). The data are particularly

S1 Fig. Data samples. Twelve randomly sampled ECG traces to illustrate the data and demonstrate the impact of pathologies on the waveform.

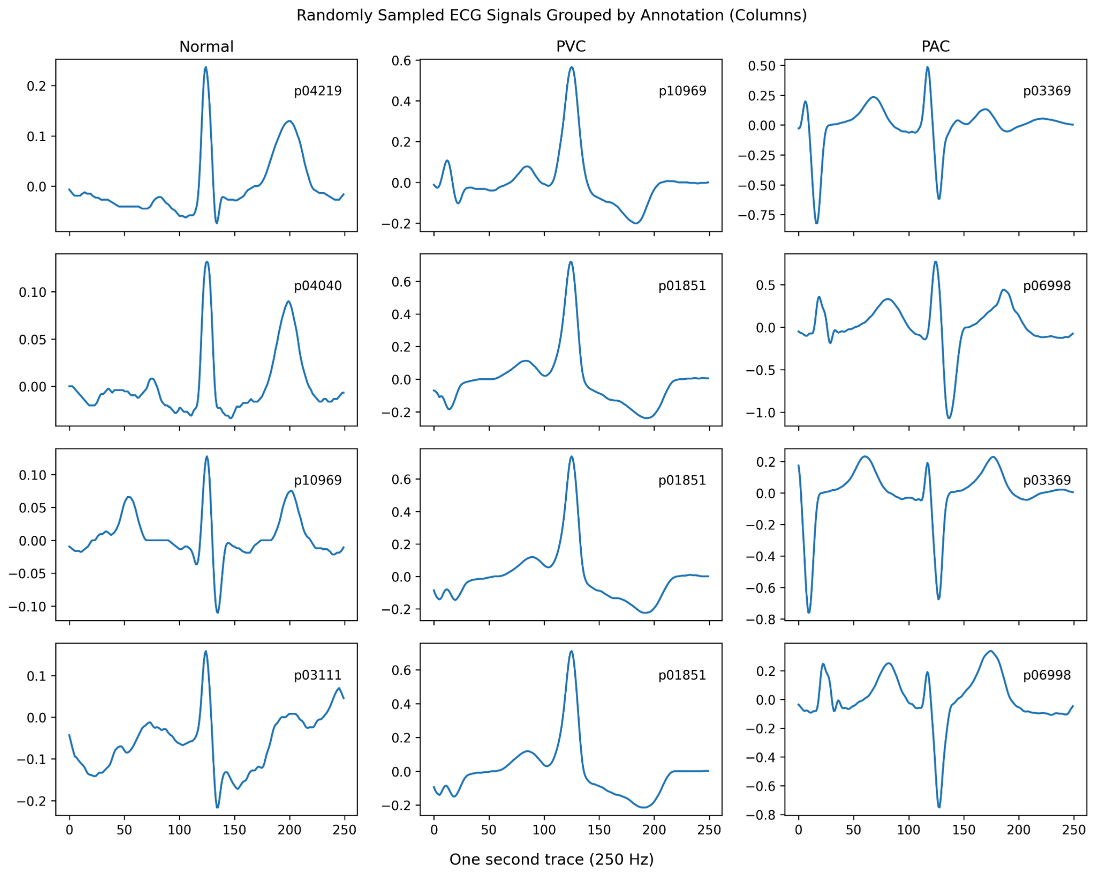

Reviewer #8

- [x] *The manuscript effectively introduces the novel application of federated unsupervised learning for anomaly detection in single-lead ECG signals, leveraging decentralized data on consumer devices. The background provides a comprehensive overview of the challenges and potential of this approach. However, it could benefit from a deeper exploration of the limitations in existing methods for ECG anomaly detection, particularly in unsupervised contexts. This would better situate the study's contributions within the broader research landscape.*

Dear Reviewer,

Thank you for acknowledging the novelty of our research and recognizing the comprehensive background provided. We agree that a deeper exploration of the limitations in existing methods contributes to the clarity of the overall objective. In response to your comment, we have added a paragraph to Section 1.3 (Research Question) to explicitly mention these gaps, which subsequently also motivate our research questions.

1.3 Reaserch Questions

The gap identified in previous research is primarily the dependence on clinically recorded ECGs (e.g., the MIT-BIH dataset)~~for most experiments, such as the MIT-BIH dataset~~, the lack of application in a consumer device setting, and few investigations into federated unsupervised learning (cf. Table 1). Although unsupervised ECG anomaly detection was successfully applied in previous studies, its implementation in the emerging domain of wearable devices remains underexplored [46]. In addition to the challenges posed by the integration of unsupervised learning with resource-constrained wearable devices, the complexity of non-IID data and data privacy requirements must be considered. Federated learning has proven to be effective for supervised tasks as a means of preserving privacy [11]; however, to the best of the authors' knowledge, few studies have investigated its application in an unsupervised setting for ECG anomaly detection. Moreover, these few studies were performed in simulated environments [40,43] or on non-consumer devices [41], limiting their generalizability to real-world outpatient scenarios. Therefore, this work focuses on the implementation of a

- [x] *The methodological framework is robust, detailing the use of a linear autoencoder model within a federated learning pipeline. The integration into a mobile application is a significant strength, demonstrating real-world applicability. However, more explicit details on ECG data preprocessing, such as segmentation and normalization, would enhance clarity. Additionally, the demographic skew in the Icentia11k dataset should be discussed to address potential biases.*

In response to your comment, we added clarifying details and supporting information about the ECG signal preprocessing.

transparency and controlled execution. The segment ECG functionality uses the *PeakSwift* package [50] to preprocess the signal as follows: (1) Detect R-peaks using the Neurokit method presented in *Makowski et al. (2021)* [51]; (2) Smooth the signal (Butterworth filter of order 5 and low cut frequency of 0.5 followed by powerline filtering) provided as a by-product of Neurokit's R-peak detection; (3) Segment~~Cut~~ the signal into one-second windows~~segments~~ around the R-peak (i.e., 500 milliseconds or 125 samples to each side) to extract single heartbeats, which are the subject of this study; (4) Upsample the segmented signals~~heartbeat segments~~ to a length of 500 by linear interpolation; (5) Map the values of the upsampled signals into the $[0, 1]$ range using min-max normalization; (6) Assign to each ECG trace a quality score by applying the method of *Zhao et al. (2018)* and following their class definitions and terminologies: *Excellent*, *Barely acceptable*, and *Unacceptable* [52]. If annotations are present, the

Because the dataset only includes aggregate statistics and lacks detailed demographic metadata, we have acknowledged this limitation in the Discussion section. In response to your comment, we explicitly noted that demographic skew may introduce additional bias into the analysis.

The demographic composition of the dataset, particularly the overrepresentation of participants in the later stages of life, present a limitation to the generalizability of the findings. Additionally, the twenty subjects randomly sampled from the large pool of 11, 000 participants may not accurately reflect the overall demographics and may introduce additional bias in the analysis. However, since the dataset did not provide demographic information on an individual subject basis, only general statements can be made about the demographics of the selected samples.

☑ *The results section is thorough, with impressive sensitivity and specificity metrics. However, the discussion should address the high false positive rate, particularly in mobile health applications where user trust is critical. An analysis of factors contributing to false positives, such as signal noise, would provide valuable insights for improvement.*

Thank you for highlighting the strong evaluation metrics in our manuscript. We agree that analyzing factors contributing to lower accuracies is crucial for user trust and for clarifying our findings. Because the dataset provides limited metadata on a sample basis, a thorough statistical analysis of variables such as age or weight on false positives was not possible. However, we identified signal noise as a significant factor and reran our evaluation using only signals rated as excellent (based on Zhao et al. 2020). We compiled these findings – along with future perspectives on addressing this issue – in the Discussion/Anomaly Detection Section. Additionally, in response to your suggestion, we explicitly emphasize the importance of user trust in self-supervised environments.

However, the system tends to categorize normal ECGs as pathological. This tendency can be explained by the unbalanced dataset (cf. S3 Fig), which is common in ECG research, and artifacts in the recorded ECGs that arise from recording in a mobile environment, causing motion noise, such as when standing up or during other physical activities. This noise caused by artifacts affects the system's ability to distinguish between real pathological and normal ECGs. 58.84% of the false-positive signals were classified as signals of *barely acceptable* quality [52], supporting the hypothesis that low-quality signals with a high signal-to-noise ratio impair the analysis.

A follow-up task is to detect movements using the IMU (Inertial Measurement Unit) sensors to identify rapid accelerations that may distort the ECG data and exclude these samples from the analysis. This approach can reduce noise and improve the reliability and trust of the anomaly detection system, which is an important factor in self-supervised environments. The high sensitivity, but also the large number of false positives, motivates to consider the presented system as a tool for filtering the predicted healthy samples and then apply computationally more demanding algorithms to distinguish true anomalies from noisy but healthy samples.

☑ *The conclusion succinctly summarizes the study's contributions, highlighting federated unsupervised learning's potential in mobile health. However, it would benefit from explicit articulation of broader implications for mobile health and anomaly detection. A forward-looking perspective on future research directions, including clinical validation and real-world deployment, would strengthen the conclusion.*

> Thank you for suggesting a forward-looking perspective and placing our work in a broader mobile health context. In response, we have revised the concluding paragraph to address these points.
>
> environment. Future research on clinical validation and deployment in real-life scenarios is needed to further validate the feasibility of the proposed system and clarify open questions, such as its performance with a growing number of participants. The investigation of more complex autoencoder models could also capture a broader range of pathologies.
>
> Overall, this study addressed important topics in mobile ECG diagnostics, including limited annotations, privacy requirements, and non-IID data. Adapting the decentralized approach to other use cases in healthcare could open up additional data sources, such as personal electronic health records on mobile devices. These are widely available but rarely released as open-source datasets. A privacy-preserving, decentralized system that employs unsupervised learning on electronic health records could enable anomaly detection across multiple health domains, particularly benefiting people in regions with limited healthcare access.
